# Comparative Analysis of LC-ESI-IM-qToF-MS and FT-NIR Spectroscopy Approaches for the Authentication of Organic and Conventional Eggs

**DOI:** 10.3390/metabo13080882

**Published:** 2023-07-25

**Authors:** Henri Lösel, Johannes Brockelt, Florian Gärber, Jan Teipel, Thomas Kuballa, Stephan Seifert, Markus Fischer

**Affiliations:** 1 Hamburg School of Food Science, Institute of Food Chemistry, University of Hamburg, Grindelallee 117, 20146 Hamburg, Germany; henri.loesel@uni-hamburg.de (H.L.); johannes.brockelt@uni-hamburg.de (J.B.); florian.gaerber@uni-hamburg.de (F.G.); stephan.seifert@uni-hamburg.de (S.S.); 2 Chemisches und Veterinäruntersuchungsamt (CVUA) Karlsruhe, Weissenburger Strasse 3, 76187 Karlsruhe, Germanythomas.kuballa@cvuaka.bwl.de (T.K.)

**Keywords:** authentication, egg, FT-NIR, LC-MS, SMD, organic vs. conventional

## Abstract

The importance of animal welfare and the organic production of chicken eggs has increased in the European Union in recent years. Legal regulation for organic husbandry makes the production of organic chicken eggs more expensive compared to conventional husbandry and thus increases the risk of food fraud. Therefore, the aim of this study was to develop a non-targeted lipidomic LC-ESI-IM-qToF-MS method based on 270 egg samples, which achieved a classification accuracy of 96.3%. Subsequently, surrogate minimal depth (SMD) was applied to select important variables identified as carotenoids and lipids based on their MS/MS spectra. The LC-MS results were compared with FT-NIR spectroscopy analysis as a low-resolution screening method and achieved 80.0% accuracy. Here, SMD selected parts of the spectrum which are associated with lipids and proteins. Furthermore, we used SMD for low-level data fusion to analyze relations between the variables of the LC-MS and the FT-NIR spectroscopy datasets. Thereby, lipid-associated bands of the FT-NIR spectrum were related to the identified lipids from the LC-MS analysis, demonstrating that FT-NIR spectroscopy partially provides similar information about the lipidome. In future applications, eggs can therefore be analyzed with FT-NIR spectroscopy to identify conspicuous samples that can subsequently be counter-tested by mass spectrometry.

## 1. Introduction

In recent years, organic husbandry is becoming more important, for example for the production of organic chicken eggs. In particular, more attention is focused on animal welfare and organic breeding methods and more environmentally friendly conditions [1,2,3,4]. In the European Union, there are different types of husbandries, which in the case of laying hens are divided into three systems: free range, barn and organic. The husbandry and the marketing of hen eggs are regulated in the European Union in Regulation (EU) No. 1308/2013, Regulation (EC) No. 589/2008, Directive 2002/4/EC and Directive 1999/74/EC. Regulation (EC) No. 2018/848 regulates the specific requirements for organic animal husbandry, animal breeding and feeding. Accordingly, the production of organic eggs is subject to strict specifications that ensure a high level of animal welfare. Organic feed may only consist of organically grown ingredients, while the addition of synthetic carotenoids to influence the color of the egg yolk is permitted in conventional feed [5,6,7,8].

Due to legal requirements, organic husbandry is more expensive compared to conventional husbandry, which is why organic products have to be marketed at higher prices [3,9]. Since consumers are willing to accept this higher price, organic egg production is growing steadily in the European Union [4,10]. However, it is not possible to visually identify the husbandry of an undamaged egg, which is why a false declaration is possible without being noticed. Especially in recent years, several cases of relabeled conventional eggs have been reported in the European Union [11,12,13,14]. In addition, in recent years it has become evident that food fraud involving organic food is the main target and has increased worldwide [15]. It has already been shown in China that eggs from different breeding systems are subject to food fraud, which can have a negative impact on egg quality, safety, fair competition and sustainable consumer confidence [16]. Accordingly, it is important that objective methods will be developed to combat food fraud worldwide, including countries with a low-trust food chain system such as in Africa or Asia.

The potential and limitations of metabolomics-based approaches for the discrimination of husbandries of animal-based food have already been demonstrated in several studies. It has been shown that polyunsaturated fatty acids (PUFA) and omega-3-fatty acids especially are more concentrated in organic meat and sheep milk [17,18]. In addition, several studies have already focused on the differentiation of the production method of eggs using different analytical strategies, including carotenoid profiling, stable isotope analysis, elemental composition, fatty acid profiling and fluorescence excitation spectroscopy [2,19,20,21,22,23,24,25]. Furthermore, UV-VIS-NIR spectroscopy showed potential to authenticate eggs according to their husbandry system [26,27].

The aim of this study was to develop an analytical strategy to discriminate and to analyze the main differences between the husbandry conditions of chicken eggs. To this end, high resolution LC-ESI-IM-qToF-MS lipidomic profiling and FT-NIR spectroscopy were applied as a low-resolution screening approach to 270 egg samples and the data obtained were analyzed by machine learning methods, in particular random forest (RF) methods. RF is based on multiple binary decision trees, each based on a different bootstrap sample containing about 63% of the samples. Therefore, for each tree, 37% of the samples, called out-of-bag (OOB) samples, remain to be evaluated and independent error estimates (OOB errors) are obtained, which do not require any further data [28]. Other advantages of RF are its very good suitability for high-dimensional data and low risk of overfitting [29,30]. To select important variables and to analyze the differences between the husbandries, surrogate minimal depth (SMD) was used [31]. SMD differs from other variable selection techniques since the variables are not evaluated individually, but as interacting groups. This is achieved by the combination of surrogate variables, originally introduced to deal with missing predictor variables, with the importance measure being minimal depth, which assess variables by their first appearance in the decision trees [32]. Furthermore, in addition to analyzing the importance of the variables, SMD can also be applied to analyze their relationship based on the mean adjusted agreement parameter. This parameter evaluates the mutual influence of variables on the classification model and has already been successfully applied to FT-NIR spectroscopy and LC-MS data [33,34]. The impact of dyes (carotenoids) for distinguishing the husbandry type was additionally investigated with photometry. Finally, to verify whether LC-MS and FT-NIR spectroscopy provide complementary or corresponding information for classification, low-level data fusion was applied and SMD was used to analyze relations across the datasets of the two analytical techniques.

## 2. Materials and Methods

### 2.1. Reagents and Chemicals

Demineralized water was ultra-purified using a Direct-Q 3 UV-R system (Merck, Millipore, Darmstadt, Germany). Isopropanol (LC-MS grade), ß-Carotin, Canthaxanthin (trans) and trans-ß-Apo-8′-carotenal were purchased from Merck KgaA (Darmstadt, Germany) and chloroform (HPLC grade), methanol (LC-MS grade) and ammonium formiate (LC-MS grade) were obtained from Carl Roth GmbH and Co. KG (Karlsruhe, Germany). Lutein and Zeaxanthin were obtained from Extrasynthese (Genay, France).

### 2.2. Samples

Sample acquisition was performed by the Chemical and Veterinary Investigation Office (CVUA), Karlsruhe [35]. A total of 270 egg samples were analyzed, which had already been analyzed by NMR spectroscopy as part of a previous study [35]. Most of the 270 egg samples came from Baden-Württemberg and were taken by official food inspectors. A smaller proportion originated from other federal states in Germany and neighboring countries. A total of 92 organic, 105 barn and 73 free-range-produced egg samples were analyzed. For statistical analysis, the barn and free-range samples were combined as conventionally produced eggs. An overview of the detailed sample information has already been published elsewhere [35].

#### Sample Preparation

The sample acquisition and processing were performed by the CVUA [35]. One sample consisted of 6 to 10 eggs, each provided by a farm in the form of one or two egg boxes. First, the egg was separated into yolk and white and both parts were mixed vigorously. The egg white was stored at −20 °C and not used for other analytical purposes. A total of 30 g of the yolk was lyophilized and then stored at −20 °C.

### 2.3. LC-ESI-IM-qToF-MS Data Acquisition

For the analysis of nonpolar compounds, 50.0 ± 0.5 mg of the 270 lyophilized egg yolk samples was weighed in a 1.5 mL reaction tube (Eppendorf, Hamburg, Germany), mixed with 1 mL of ice-cold extracting solvent [chloroform/isopropanol (1:2, *v*/*v*)] and two steel balls (⌀ = 3.25 mm). Subsequently, the samples were ball-milled for 3 min at 3 m/s using a Bead Ruptor 24 equipped with a 1.5 mL microtube carriage kit (Biolabproducts, Bebensee, Germany). The remaining suspension was centrifuged for 5 min at 14,000× *g* and at 4 °C (Sigma 3-16PK, Sigma, Osterode, Germany). The supernatant was centrifuged again for 20 min under the same conditions to ensure that proteins were completely removed. For analysis, the supernatant was diluted to a 1:4 (*v*/*v*) ratio using the extraction solvent. To ensure that the signals identified in the egg samples were not present as contaminants in the instrument, chemicals or consumables, an extraction blank was also processed analogously. Each extract was measured on the day it was made, as well. In addition, a pooled extract was prepared by mixing 10 μL of each of the 270 egg yolk extracts and was used for MS/MS measurements.

A 6560 Ion Mobility qToF LC-MS system was used for sample analysis (Agilent Technologies, Santa Clara, CA, USA). Liquid chromatography was performed with an Agilent 1290 Infinity II UHPLC System equipped with a high-speed pump (G7120A, 1290 high-speed pump), multisampler (G7167B, 1290 multisampler) and a temperature-controlled column compartment (G7116B, 1290 MCT). Non-polar metabolites were separated with an Accucore RP-MS UPLC column (150 mm × 2.1 mm i.d., 2.6 μm) equipped with a guard column of the same material (10 mm × 2.1 mm, i.d., 2.6 μm) (Thermo Fisher Scientific, Braunschweig, Germany).

To check the reproducibility, a quality control (QC) sample (randomized chosen sample) was injected every ten measurements. Every five measurements a blank sample was analyzed where no injection was done. Samples were measured in random order, taking into account the drifts of the instrument. The autosampler was tempered to 5 °C. To ensure constant conditions, the column oven was set to 30 °C, with a flow rate of 300 μL/min. The mobile phase was water and isopropanol/methanol (3:1, *v*/*v*), with the addition of 0.1 mM ammonium formate at pH 3.5. The chromatography gradient is shown in Table S1 in the Supplementary Materials. The injection volume was set to 2 μL.

Analysis was performed in positive ionization mode in a mass range from 50–1700 *m*/*z* with the following settings: gas temperature, 225 °C; drying gas flow rate, 10 L/min; nebulizer, 40 psi; sheath gas temperature, 375 °C; sheath gas flow rate, 12 L/min; and capillary voltage, 3000 V. The IMS parameter were drift gas, nitrogen; drift gas pressure, 3.95 Torr; frame rate, 0.9 frame/s; IM transient rate, 18 IM transients/frame; max. drift time, 60 ms; TOF Transient rate, 600 Transients/IM transients; trap fill time, 3900 μs; trap release time, 250 μs; and multiplexing pulse sequence length, 4 bit. Drift times were calibrated by infusing the Agilent Technologies ESI tuning mix (Agilent Technologies, Santa Clara, CA, USA, part number: G1969-85000) and Hexamethoxyphosphazine under the same conditions for 1 min, once a day.

The instrument was calibrated immediately before the series of measurements with the tuning mix. Furthermore, a lock mass calibration was conducted using a second sprayer during measurements using purine and Hexakis(1H, 1H, 3H-tetrafluoropropoxy)phosphazine.

MS/MS spectra were recorded for the most important marker substances. Identification was based on the high-resolution mass and fragment spectra and partially supported by the Lipid Annotator software (Agilent Technologies, Santa Clara, CA, USA) as well as by the database LipidMaps [36]. In addition, the CCS values were compared with the LipidCCS database [37].

### 2.4. MS Data Processing

A PNNL Preprocessor (version 2020.03.23) was used for the demultiplexing of IM-TOF data files with the following parameters: demultiplexing checked; moving average window size 5 frame; moving average smoothing checked; *m*/*z* not used; drift 3; chromatography/infusion 3, and signal intensity lower threshold 20 counts. The calibration of CCS values was carried out with IM-MS Browser software (version 10.0). Four-dimensional feature finding was performed with Mass Profiler software (version 10.0) with the following parameters: restrict RT to 0.0–30.0 min; ion intensity > 150.0 counts; isotope model common organic (no halogens); limit charge states to a range of 1–2; report single-ion features with charge state z = 1; RT tolerance = ±10.0% + 0.50 min; DT tolerance = ±1.5%; mass tolerance = ±20.0 ppm + 2.0 mDa; and Q-Score > 70.0. The bucket table was exported as an .xls file. A bucket had to be detectable in at least 50% of all samples from one sample group (organic and conventional), leaving 1727 variables for statistical analysis. Vector normalization and autoscaling were performed.

### 2.5. Fourier Transform near Infrared Spectroscopy

A total of 1.3 ± 0.1 g of the 270 lyophilized egg yolk samples was weighed directly into glass vials (52.0 mm × 22.0 mm × 1.2 mm, Nipro Diagnostics Germany GmbH, Ratingen, Germany) and measurements were performed at 22 ± 2 °C. The glass vials were shaken thoroughly, levelled out uniformly and used directly for the FT-NIR spectroscopy analysis by using an FT-NIR spectrometer from Bruker with an integration sphere used, equipped with OPUS software (TANGO, Bruker Optics, Bremen, Germany). The spectrum was recorded in reflectance mode with 50 scans in a wavenumber range of 11,550–3950 cm^−1^ and a resolution of 4 cm^−1^. Five technical replicates were measured per sample.

### 2.6. FT-NIR Spectra Preprocessing

Preprocessing was performed using MATLAB R2021b (The MathWorks Inc., Natick, MA, USA). Different particle sizes of the sample material led to so-called scattered light effects. Therefore, first, multiplicative scattered light correction (MSC) was used to normalize the data and reduce effects such as additive and multiplicative scattering [38]. In the next step, the first derivative of the spectra was calculated, reducing offsets and other additive effects [39,40]. Smoothing was then performed using a Savitzky–Golay filter, which can improve the signal-to-noise (S/N) ratio [41]. Binning of five adjacent wavenumbers was performed, reducing the number of variables (3720 original variables), leaving 744 variables for data analysis, and decreasing the calculation time [34,42]. Since in an FT-NIR spectrum contiguous wavenumbers correlate strongly with each other, this makes the data easier to handle without loss of information [43]. In the last step, the median of the five spectra of one sample was calculated.

### 2.7. Photometry

A total of 200 μL of the undiluted extracts from each of the 270 egg yolk samples of the LC-MS analysis were pipetted in a 96-well microtiter plate for photometric analysis. The data were acquired with a SpectraMax M5e instrument (Molecular Devices, LCC, San Jose, CA, USA) equipped with the software SoftMax Pro 6.1 (Molecular Devices, LCC, San Jose, CA, USA). A photometric spectrum in a wavelength range of 380–790 nm with a resolution of 1 nm was acquired, resulting in 411 variables for statistical analysis.

### 2.8. Multivariate Data Analysis

Principal component analysis (PCA) was performed with the software MATLAB R2021b. For the application of random forest approaches, the R packages ranger in version 0.14.1 [44] and Surrogate Minimal Depth in version 0.2.0 [31] were applied in R version 4.2.2 to the processed LC-MS (1727 variables), photometry (411 variables) and FT-NIR spectroscopy (744 variables) datasets separately, with the parameters ntree = 10,000 and minimal.node.size = 1. In addition, mtry was set to 267, 142 and 91 for the LC-MS, FT-NIR spectroscopy and photometry dataset (corresponding to the 3/4 power of the total variables), respectively. To compensate for the class imbalance, the parameter case.weights was chosen accordingly, meaning that the weights of the samples of the smaller class (organic) were weighted higher and the samples of the larger class (conventional) were weighted lower. The weight of each class was therefore determined by dividing the size of the largest class by the size of the respective class. For SMD, the number of surrogate variables was set to 87 for LC-MS and 149 for FT-NIR spectroscopy, corresponding to 5 and 20% of the total variables of the LC-MS and FT-NIR dataset, respectively. The value for s was chosen as in previous applications and was higher for the FT-NIR data, since the variables show higher collinearity [33,34]. The LC-MS and FT-NIR spectroscopy datasets were then merged and analyzed using the same parameters as for the individual datasets, but with an mtry of 350 and 124. For visualization of the relation parameter mean adjusted agreement, a heatmap was created using the R package pheatmap [45].

## 3. Results and Discussion

### 3.1. LC-ESI-IM-qToF-MS

The first objective of this study was to analyze the 270 egg samples with LC-ESI-IM-qToF-MS with the aim of determining differences between the husbandry systems of chicken eggs. To evaluate the reproducibility of the LC-MS analysis, the preprocessed data containing 1727 variables of the 270 egg samples and the 28 measurements of the QC sample were analyzed with PCA. Based on the scores, the QC samples showed little scatter, so that the measurements were reproducible (Figure S1 in the Supplementary Materials). An exemplary total ion chromatogram of a pooled extract from all 270 egg samples with an assignment of the main substance classes (Lyso-glycerophosphocholines, phosphocholines, sphingomyelins, triacylglycerols) is shown in Figure S2 in the Supplementary Materials.

**Figure 1:**
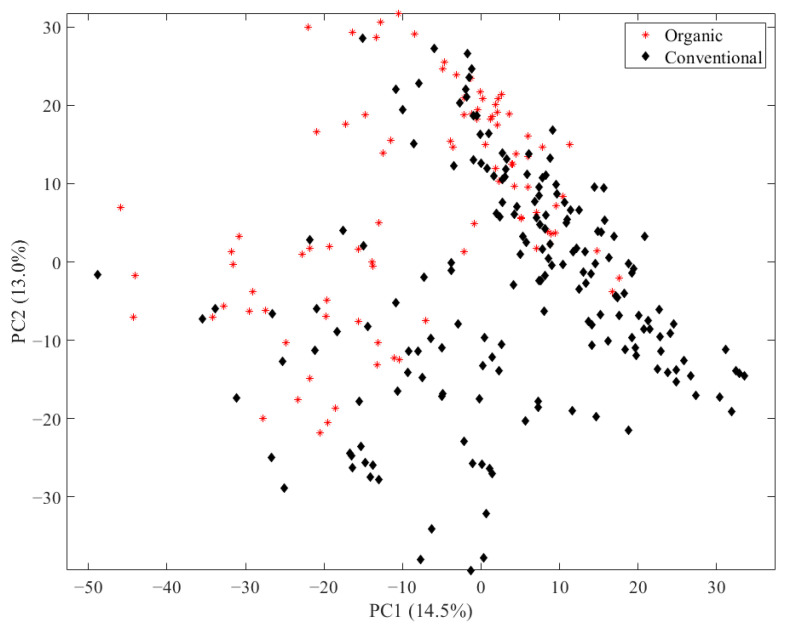
Scores of the first two principal components of the PCA applied to LC-MS data of 270 egg samples depicted according to their husbandry (organic husbandry as red stars; conventional as black diamonds).

In order to analyze the main differences in the LC-MS data of the egg yolk, PCA was performed and the scores of the first two principal components, together representing 27.5% of the total variance, were depicted according to their husbandry (Figure 1). PC1 and PC2 combined represent only a small amount of the total variance of the data set. Nevertheless, for PCAs with LC-MS data and a high number of variables that are not correlated but independent, it often occurs that the PCs only show low variances [46,47,48]. Only a few samples from conventional husbandry are located in the lower right corner of the scores plot and show no overlap with samples from organic husbandry. However, there is no clear separation between organic and conventional samples with the unsupervised approach PCA, which is why the supervised approach random forest was applied subsequently with all 1727 variables.

The confusion matrix of the classification of the 270 egg yolk samples with random forest is shown in Table 1 in percentage. The classification result implies that it is possible to distinguish the husbandry type of chicken eggs by LC-MS. Only 2.2% (2 samples) of the organic samples were misclassified, while only 4.5% (8 samples) of the conventional eggs were classified as organic.

In order to analyze the differences between the husbandry types in more detail and to select important variables, a total of 211 variables were selected (see Table S2 for a list of selected variables together with their SMD score). From this group, a total of 33 variables were identified based on their MS/MS spectra, including six triacylglycerols (TAG) identified as [M + Na]^+^ and [M + NH_4_]^+^ adducts, ultimately leaving 27 identified marker compounds. The classes of identified lipids included carotenoids and TAGs (see Table S3 for a list of all selected marker compounds with identification parameters).

**Figure 2:**
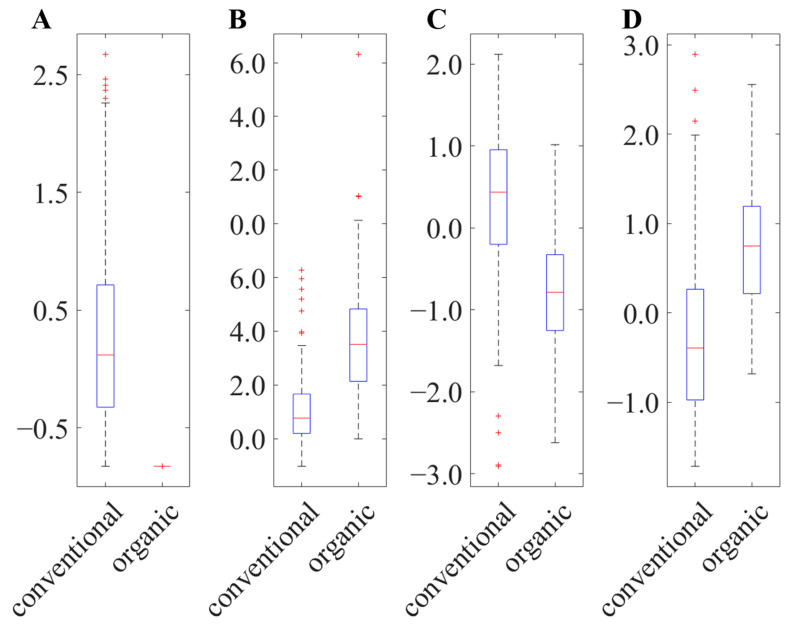
Boxplots of the normalized signal intensity of (**A**) canthaxanthin, (**B**) lutein/zeaxanthin, (**C**) TAG (18:1/16:1/16:1) and (**D**) TAG (18:3/18:2/18:1), which are chosen exemplary from the selected metabolites for the differentiation of conventional and organic eggs.

The four most important variables with the lowest SMD values (approx. 0.02) were signals at the retention times of 7.4 min and 7.7 min, both of which could be divided into two different signals due to different drift times and thus different CCS values. Based on these values and additional MS/MS investigations in connection with the analysis of reference samples (compare Figures S3 and S4), the selected variables could be assigned to canthaxanthin, showing elevated concentrations in conventional samples and values at 0 for organic samples (Figure 2A). Hens are not able to biosynthesize canthaxanthin, so it is determined by the feed [49]. Adding it to the feed is not allowed in organic husbandry according to the council regulation No 2018/848. Therefore, canthaxanthin could be a useful marker for conventional husbandry, which was already shown in another study [50].

Furthermore, two additionally selected variables were identified as carotenoids that showed elevated concentrations in samples from organic husbandry (Figure 2B). These metabolites were isobaric compounds (*m*/*z*: 569.4) that eluted at a retention time of 6.5 min and could be separated by the ion mobility cell (CCS value: 291 Å and 308 Å; see Table S3 in the Supplementary Materials). The MS/MS spectrum showed a signal at 551 *m*/*z*, which identified the two signals as lutein/zeaxanthin. Since both differ only by the position of a double bond, the fragmentation patterns are comparable, which was confirmed by measurements of commercial standards (Figures S5 and S6) [51]. The color of the yolk in organically produced eggs is determined by the vegetables fed, and since mainly corn containing lutein/zeaxanthin is used, these substances are more concentrated in these eggs [52,53].

To evaluate the influence of the identified carotenoids on the classification of the eggs, the 270 egg samples and carotenoid standards were measured photometrically and the classification was conducted in the same way as for LC-MS analysis. An accuracy of 92.2% was obtained (Table S4 in the Supplementary Materials). In Figure S7, the spectra of the averaged samples from organic and conventional husbandry are compared and the absorbance of the organic samples in the range from 430–450 nm is higher and flattens out faster after the absorption maximum at 480 nm than for the conventional samples. In contrast, the spectra of the conventional samples show comparatively high absorption at wavelengths above 500 nm. The analysis of the carotenoid standards shows an absorbance maximum around 480 nm for canthaxanthin, while lutein/zeaxanthin have their absorbance maxima around 450 nm (Figure S8 in the Supplementary Materials). In summary, these results demonstrate that carotenoids have a significant influence on the differentiation of the husbandry conditions of conventionally and organically produced eggs. Although the classification results with photometry are promising and this method has potential to be used as a rapid screening method, its suitability is limited since the carotenoid profile can be easily influenced by the feed [54].

In addition to carotenoids, the selected variables could also be assigned to lipids (mainly TAGs, which ionized as [M + Na]^+^ and [M + NH_4_]^+^ adducts). TAGs were identified by the fragments that result from the cleavage of a fatty acid and the neutral loss of this fatty acid [55]. In addition, [RCOO + 58]^+^ fragments were used for structural elucidation [56]. A total of 21 different lipids were identified (Table S3 in the Supplementary Materials). Figure 2C,D shows boxplots of the exemplary chosen TAG (18:1/16:1/16:1) and TAG (18:3/18:2/18:1). It is apparent that the identified lipids containing polyunsaturated fatty acids (PUFA) have an increased concentration in organic eggs (Figure 2D), while conventionally produced eggs contain lipids with saturated or monounsaturated fatty acids (Figure 2C; see Table S3). These findings are consistent with results of NMR analysis, where signals from PUFAs were increased in organically produced samples [35]. Mugnai et al. showed that PUFA concentrations are increased in organically produced eggs because hens have more access to outdoor space and for this reason have more access to grass [21].

In summary, carotenoids and lipids were selected as possible marker substances to distinguish eggs from different husbandries by LC-MS. The reason for the differences in carotenoid composition is probably due to synthetic carotenoids added to the hen feed in conventional husbandry, but not in organic husbandry [54]. It has already been shown that the fatty acid composition of the egg yolk is influenced by the feed [57], nevertheless the adjustment of a specific lipid composition is not as easily controllable as in the case of carotenoids. As egg producers with the intention of fraud could easily adapt the feed they give to hens from conventional husbandry, lipids are more suitable as marker substances for the differentiation of the husbandry.

### 3.2. FT-NIR Spectroscopy

**Figure 3:**
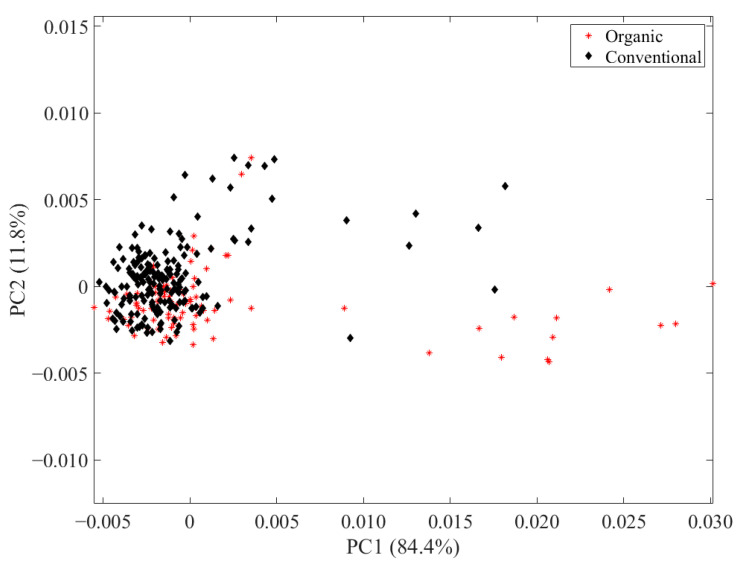
Scores of the first two principal components of the PCA applied to FT-NIR spectroscopy data of 270 egg samples depicted according to their husbandry (organic husbandry as red stars; conventional as black diamonds).

The 270 egg samples were also analyzed by FT-NIR spectroscopy as a low-resolution screening method to differentiate the husbandry of chicken eggs. First, to analyze the main differences in the FT-NIR data of the egg yolk measurements, PCA was performed on the data containing 744 variables and the scores of the first two principal components, together representing 96.2% of the total variance, were depicted according to their husbandry (Figure 3). The scores show a strong overlap of the samples from both husbandries and no differentiation is evident. For this reason, random forest was used as a supervised approach to determine differences between the husbandries.

The classification and variable selection were performed in the same way as for LC-MS analysis. The confusion matrix is shown in Table 2 in percentage.

**Figure 4:**
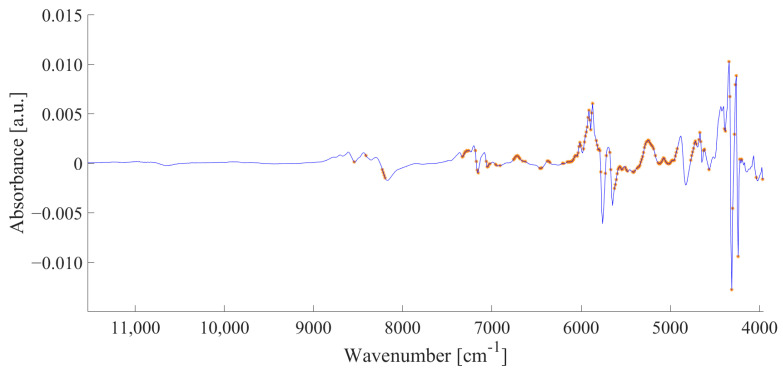
Selected variables (orange star) in the averaged NIR-Spectra of all egg yolk samples (blue line) after preprocessing (MSC, first derivative, smoothing, binning of five adjacent variables and formation of the median). A list of the selected variables is summarized in Table S5 in the Supplementary Materials. The unit of absorbance is given in absorbance unit (a.u.).

Compared to the LC-MS classification result, more samples were misclassified with the FT-NIR spectroscopy data. A total of 28.3% (26 samples) of the organic samples were assigned as conventional and 15.7% (28 samples) of the conventional samples as organic. The difference between the two classification results was to be expected, as FT-NIR spectroscopy is a low-resolution method and the achievable depth of information is lower compared to LC-MS analysis. SMD selected 166 bins from the FT-NIR spectrum, which are highlighted in Figure 4. A full list of all selected variables is given in Table S5 in the Supplementary Materials. Variables were selected in different regions of the FT-NIR spectrum that could be assigned to different molecule vibrations and thus to different substance classes, namely to lipids and proteins (SMD approx. 0.01–0.30).

Variables from several regions of the spectrum associated with lipids were selected. Most of them were in the range between 5700 and 6000 cm^−1^, assigned to the first overtone of saturated and unsaturated fatty acids. Furthermore, variables in the ranges between 4000 and 4300 cm^−1^ and 8400 and 8600 cm^−1^ could be assigned to the first overtone of C-H vibrations and the second overtone of C-H stretching from unsaturated fatty acids, respectively, while the signals at 8300 cm^−1^ are associated with the second overtone of C-H stretching vibrations [58]. In addition, several variables were selected in the range between 6000 and 7300 cm^−1^. They could be assigned to the first overtone of the N-H and O-H vibrations of the peptide bond [58].

### 3.3. Data Fusion

Low-level data fusion was performed to evaluate if the classification accuracy increases by merging the data from LC-MS and FT-NIR spectroscopy. Furthermore, SMD was applied to analyze the relations of the variables of the two analytical techniques. The confusion matrix of the merged dataset from the 270 egg samples is shown in Table S6 in percentage.

**Figure 5:**
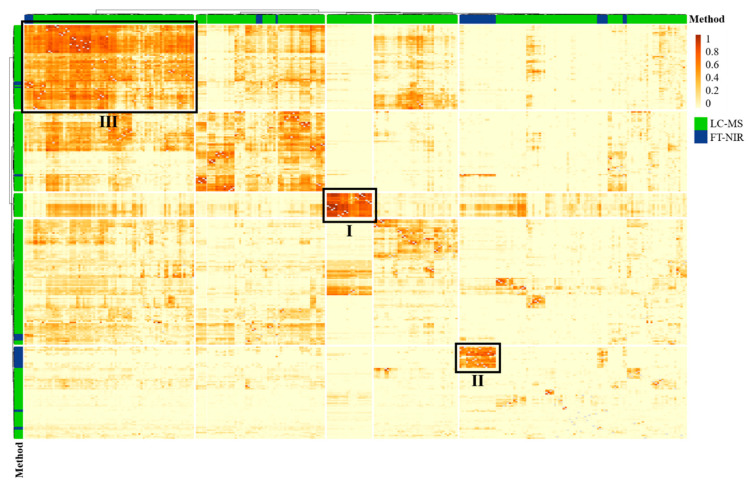
Results of the relation analysis of selected variables with SMD. A hierarchical cluster analysis using Euclidean distances and the Ward algorithm was applied to the mean adjusted agreement values, and the variables of the LC-MS and FT-NIR spectroscopy dataset are marked in green and blue, respectively. The intensity of the coloring indicates the mean adjustment agreement between the respective variables. The clusters are labeled with (**I**–**III**) and were assigned to: (**I**) carotenoids from the LC-MS analysis; (**II**) protein-associated bands of the FT-NIR spectrum; and (**III**) lipids from the LC-MS analysis and lipid-associated bands of the FT-NIR spectrum.

Both the classification accuracy of 96.3% and the misclassified samples are equal to the LC-MS analysis. Thus, no improvement of the classification can be achieved by combining the data (Table S6). However, more interesting than the classification result is the analysis of the relations between the variables of both datasets, which are shown in a heatmap in Figure 5.

In the heatmap, different types of clusters are apparent. First, there is one cluster (Cluster I) that contains only LC-MS variables that have a high relation. This cluster contains all of the identified canthaxanthin variables. This is in accordance with a previous study using SMD for the analysis of LC-MS data, in which related variables could often either be assigned to the same metabolite (e.g., different adducts) or to metabolites with meaningful biological relations (e.g., metabolic pathways) [33]. Since this cluster only contains LC-MS variables, it can be concluded that carotenoids have only little impact on the FT-NIR spectra. This assumption is supported by other studies, in which the carotenoid content of passion fruit, corn, apricot and palm oil was investigated using NIR spectroscopy [59,60,61,62]. In these studies, the determination of the carotenoid content showed high error rates, which was explained by a low intensity of the absorption bands of carotenoids compared to the bands of the main ingredients [59,62].

Cluster II contains only FT-NIR spectroscopy variables between 5900 and 6100 cm^−1^. This is due to the fact that these variables can be assigned to N-H and C=O groups of the peptide backbone, referred to as the ß-sheet structure [58]. No LC-MS variables are present in this cluster, since the proteome is not covered by the used method, as proteins are precipitated during the extraction process [63]. In a previous study in which SMD was applied to an FT-NIR spectroscopy dataset, it was already shown that neighboring variables in the FT-NIR spectrum are related. This is due to the fact that several signals are caused by the same substance [34].

Cluster III is more interesting than the clusters already mentioned because, here, both LC-MS and FT-NIR spectroscopy variables are present, implying that the variables of the two analytical approaches may be associated to the same metabolites. The FT-NIR spectroscopy variables are in the range around 5800 cm^−1^ and can be assigned to the C-H vibration of the first overtone of methylene groups in hydrocarbon chains [58]. The corresponding LC-MS variables are predominantly signals from TAGs that contain saturated, monounsaturated, and polyunsaturated fatty acids. Therefore, this cluster probably contains variables from TAGs detected by both analytical techniques.

In summary, the analysis of variable relations showed that carotenoids are only detected by LC-MS, proteins only by FT-NIR spectroscopy and lipids by both approaches.

## 4. Conclusions

In this study, high-resolution LC-ESI-IM-qToF-MS and FT-NIR spectroscopy were successfully applied to distinguish the husbandries of chicken eggs. Based on the results of a low-level data fusion, it was shown that FT-NIR spectroscopy and LC-MS partially covered the same parts of the lipidome of eggs. Therefore, FT-NIR spectroscopy could be used to indicate suspicious samples during an incoming inspection, which can then be analyzed in more detail by LC-MS to determine the husbandries. For this purpose, the identified marker lipids can be analyzed using a targeted approach, e.g., an LC-ESI-QqQ-MS instrument. SMD was successfully applied to a fused dataset for the first time and has shown promise for analyzing relations between different datasets in future studies.

## Figures and Tables

**Table 1:** Confusion matrix of the random forest for the differentiation of the husbandries of the 270 egg samples based on LC-MS data.

		**Predicted**		
		**Organic (%)**	**Conventional (%)**	**Sensitivity (%)**
True	Organic (%)	97.8	2.2	97.8
	Conventional (%)	4.5	95.5	95.5
	Specificity (%)	91.8	98.8	96.3

**Table 2:** Confusion matrix of the random forest for the differentiation of the husbandries of the 270 egg samples based on FT-NIR spectroscopy data.

		Predicted		
		Organic (%)	Conventional (%)	Sensitivity (%)
True	Organic (%)	71.7	28.3	71.7
	Conventional (%)	15.7	84.3	84.3
	Specificity (%)	70.2	85.2	80.0

## Data Availability

The datasets of the FT-NIR spectroscopy and photometry created as part of the current study are available in the Zenodo repository at: https://doi.org/10.5281/zenodo.7345560, accessed on 5 June 2023. The LC-MS dataset presented in this study is available on request from the corresponding author. The data are not publicly available due to the data size exceeding the allowable amount of common repositories.

## Supplementary Materials

The following supporting information can be downloaded at: https://www.mdpi.com/article/10.3390/metabo13080882/s1, Figure S1: Scores of the PCA in which the 28 measurements of the quality control (QC) sample and the measurements of the 270 authentic samples were depicted; Figure S2: An exemplary total ion chromatogram of a pooled egg yolk extract; Figure S3: MS/MS spectrum of the *m*/*z* ratio 565.403 at an RT of 7.7 min in an egg sample extract; Figure S4: MS/MS spectrum of a canthaxanthin standard (*m*/*z*: 565.403; RT: 7.7 min); Figure S5: MS/MS spectrum of a lutein standard (*m*/*z*: 569.434; RT: 6.5 min); Figure S6: MS/MS spectrum of a zeaxanthin standard (*m*/*z*: 569.434; RT: 6.5 min); Figure S7: Comparison of the photometry spectra of egg yolk extracts from organic (blue) and conventional (orange dashed) husbandry; Figure S8: Comparison of the photometry spectra of carotenoid standards; Table S1: Overview of the chromatography gradient; Table S2: Summary of the selected variables of the LC-MS dataset with surrogate minimal depth; Table S3: Summary of identification parameters of marker compounds that were selected by SMD; Table S4: Confusion matrix of the random forest for the differentiation of the husbandries of eggs based on photometry data; Table S5: Summary of the selected variables of the FT-NIR spectroscopy dataset with SMD; Table S6: Confusion matrix of the random forest for the differentiation of the husbandries of eggs based on the merged LC-MS and FT-NIR spectroscopy data; Table S7: Summary of the selected LC-MS variables from the merged LC-MS and FT-NIR spectroscopy dataset with SMD; Table S8: Summary of the selected FT-NIR spectroscopy variables from the merged LC-MS and FT-NIR spectroscopy dataset with SMD.
