# Supplementary material for: Comparative Analysis of LC-ESI-IM-qToF-MS and FT-NIR Spectroscopy Approaches for the Authentication of Organic and Conventional Eggs
Source: Metabolites. 2023 Jul 25;13(8):882. doi: 10.3390/metabo13080882 (PMC10456441; doi:10.3390/metabo13080882)
Supplement: Supplementary file 1 [file metabolites-13-00882-s001.zip › metabolites-2476587-supplementary.pdf]

metabolites

Supplementary Materials

Comparative Analysis of LC-ESI-IM-qToF-MS and FT-NIR Spectroscopy Fingerprinting for Distinguishing Organic and Conventional Eggs

Henri Lösel [1,†], Johannes Brockelt [1,†], Florian Gärber [1], Jan Teipel [2], Thomas Kuballa [2], Stephan Seifert [1] and Markus Fischer [1,*]

1 Hamburg School of Food Science, Institute of Food Chemistry, University of Hamburg, Grindelallee 117, 20146 Hamburg, Germany; henri.loesel@uni-hamburg.de (H.L.); johannes.brockelt@uni-hamburg.de (J.B.); florian.gaerber@uni-hamburg.de (F.G.); stephan.seifert@uni-hamburg.de (S.S.)

2 Chemisches und Veterinäruntersuchungsamt (CVUA) Karlsruhe, Weissenburger Strasse 3, 76187 Karlsruhe, Germany; jan.teipel@cvuaka.bwl.de (J.T.); thomas.kuballa@cvuaka.bwl.de (T.K.)

* Correspondence: markus.fischer@uni-hamburg.de; Tel.: +49-40-42838-4359

† These authors contributed equally to this work.

Supplementary Figures

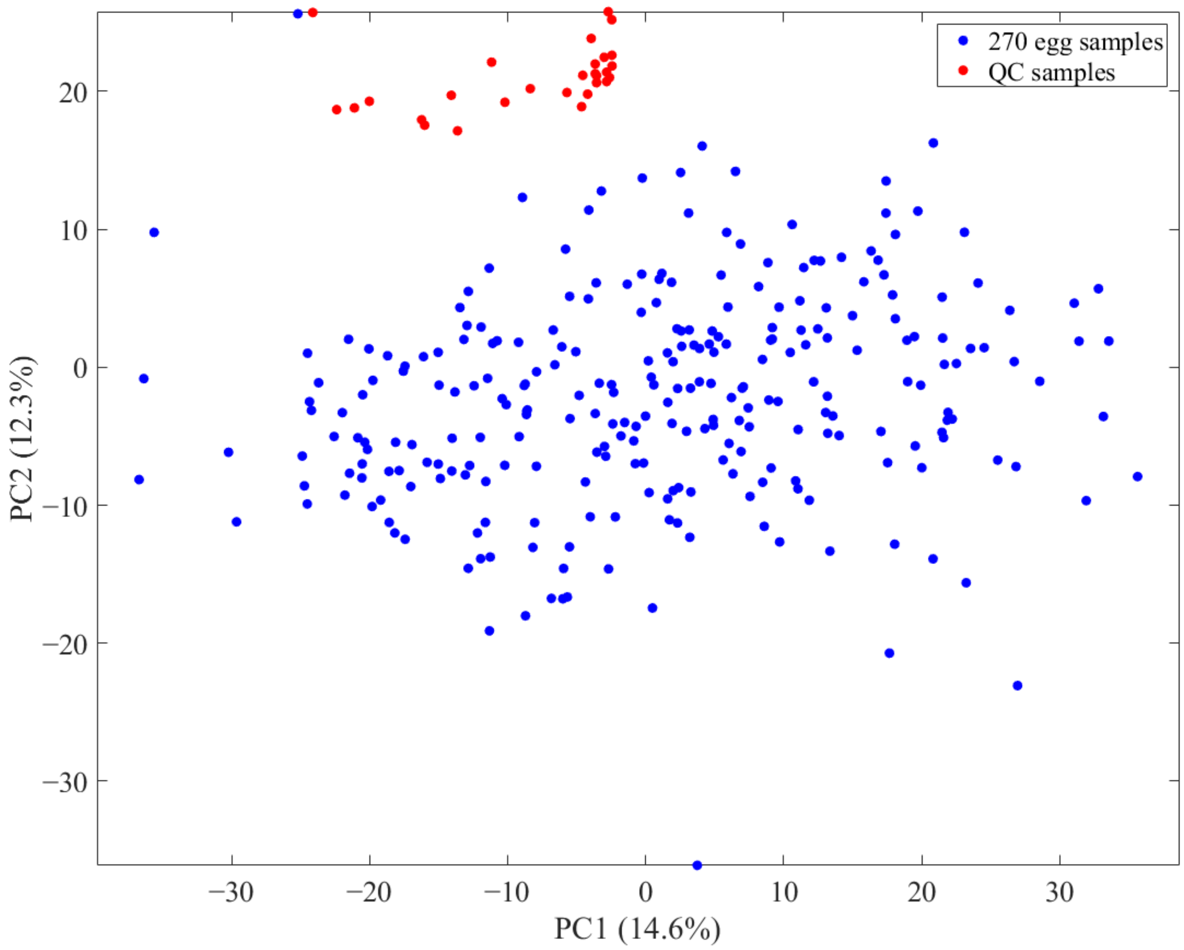

Figure S1. Scores of the PCA in which the 28 measurements of the quality control (QC) sample and the measurements of the 270 authentic samples were depicted (QC measurements as red circles, other samples as blue circles).

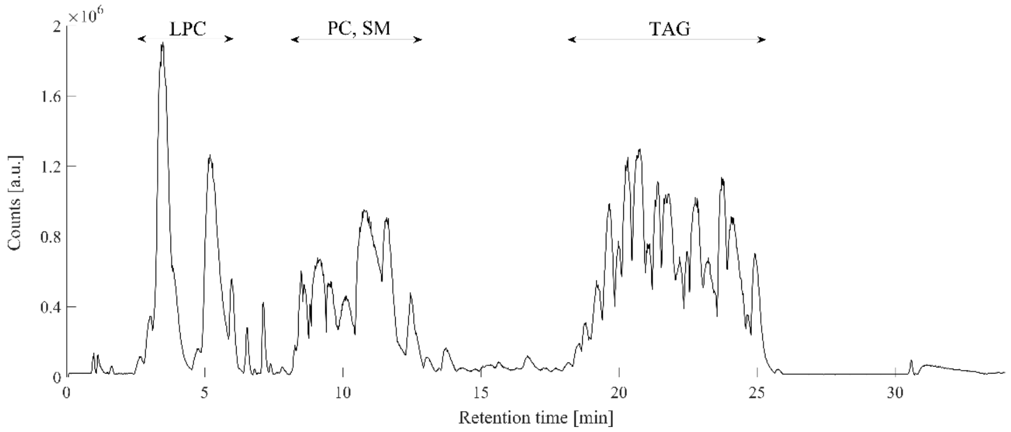

Figure S2. An exemplary total ion chromatogram of a pooled egg yolk extract. The retention time (RT) (x-axis) is plotted against the counts (y-axis). LPC: Lyso-glycerophosphocholine, PC: Phosphocholine, SM: Sphingomyelin, TAG: Triacylglycerol.

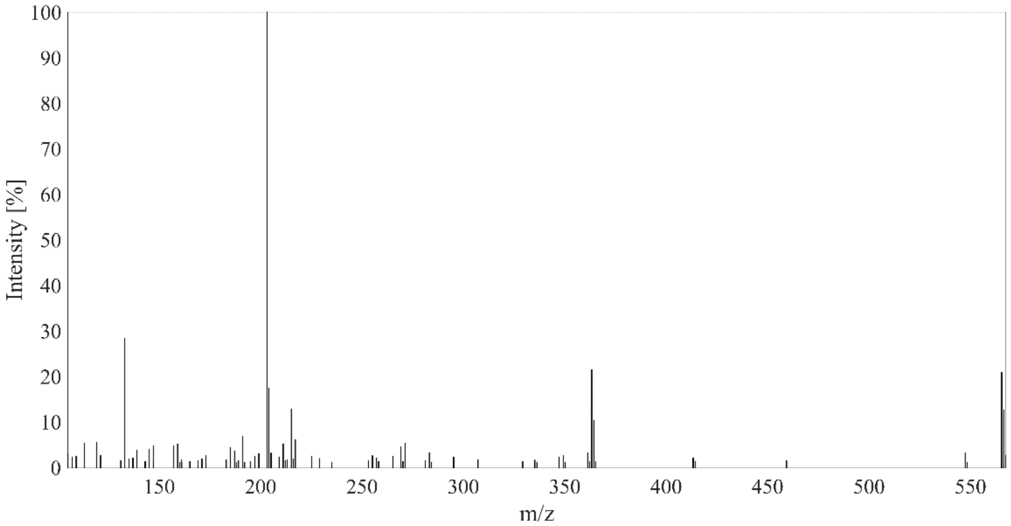

Figure S3: MS/MS spectrum of the *m/z* ratio 565.403 at a RT of 7.7 min in an egg sample extract. The collision energy was 10 eV. The compound was identified as canthaxanthin.

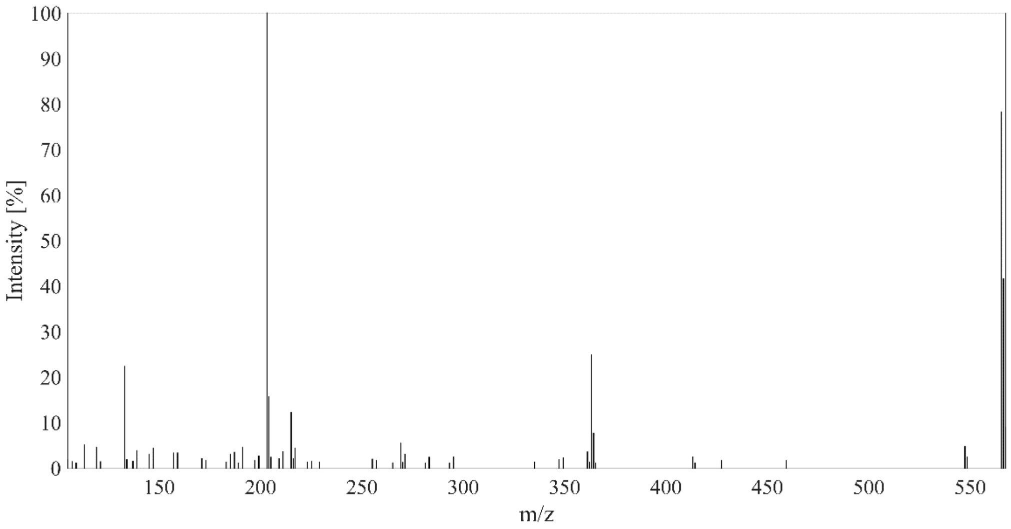

Figure S4: MS/MS spectrum of a canthaxanthin standard (*m/z*: 565.403; RT: 7.7 min). The collision energy was 10 eV.

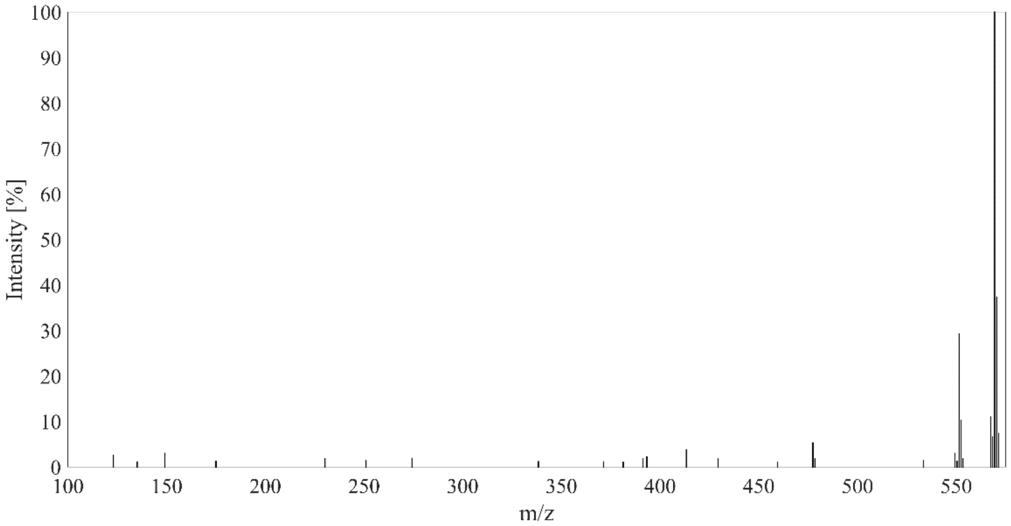

Figure S5: MS/MS spectrum of a lutein standard (*m*/*z*: 569.434; RT: 6.5 min). The collision energy was 10 eV.

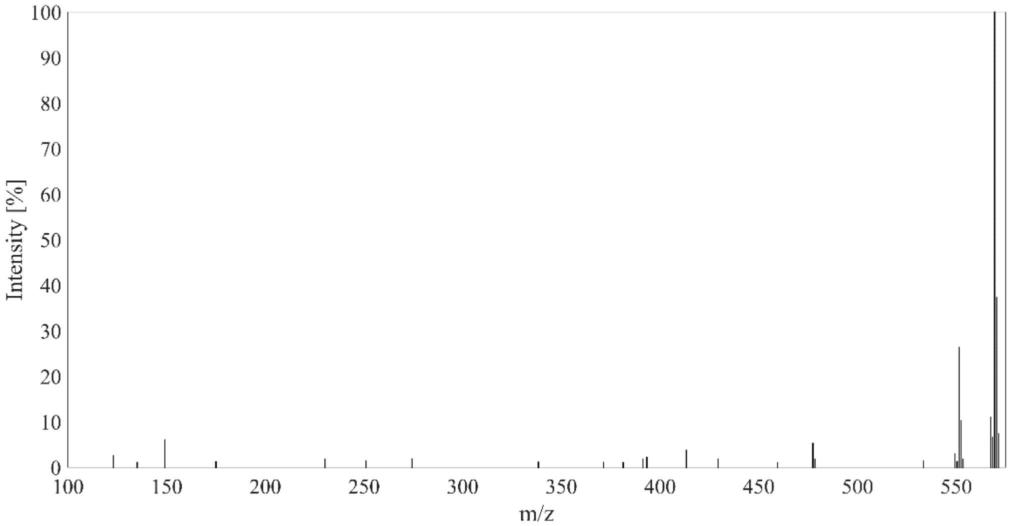

Figure S6: MS/MS spectrum of a zeaxanthin standard (*m*/*z*: 569.434; RT: 6.5 min). The collision energy was 10 eV.

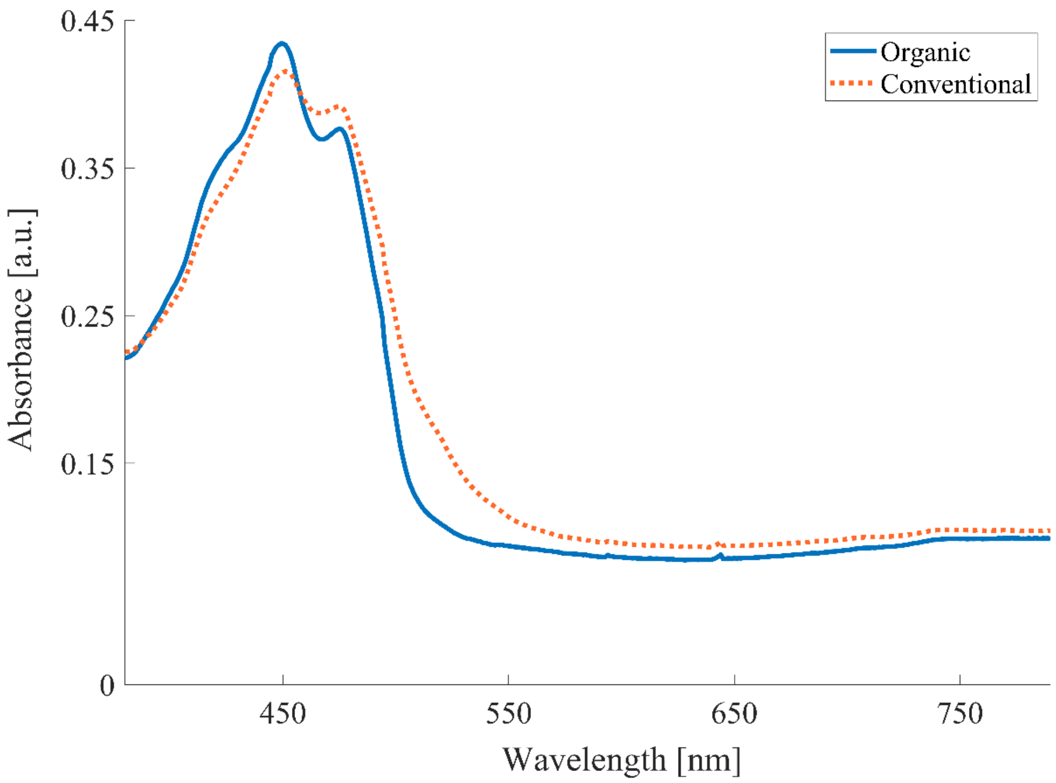

Figure S7: Comparison of the photometry spectra of egg yolk extracts from organic (blue) and conventional (orange dashed) husbandry. The individual spectra were averaged according to their husbandry. The unit of absorbance is given in absorbance unit (a.u.).

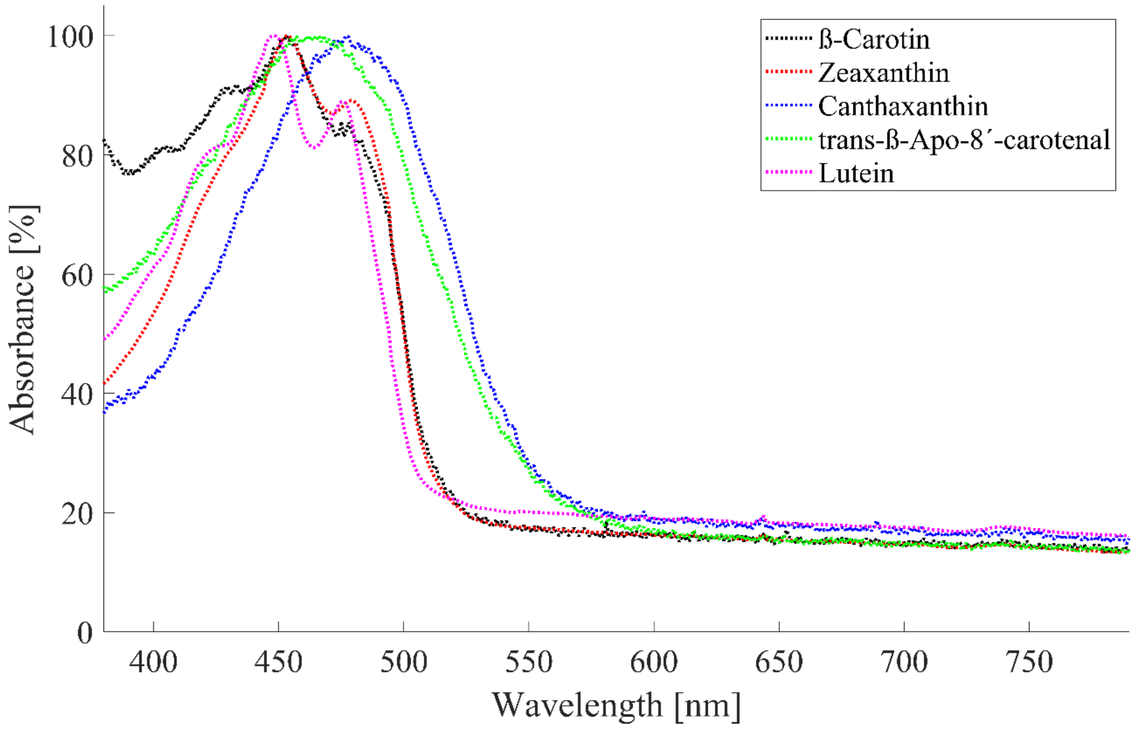

Figure S8: Comparison of the photometry spectra of carotenoid standards. The highest absorbance in the spectrum from each standard was normalized to 100% (ß-Carotin: black; Zeaxanthin: red; Canthaxanthin: blue; trans-ß-Apo-8´-carotenal: green; Lutein: pink).

Supplementary Tables

Table S1. Overview about the chromatography gradient. Both solvents had an addition of 0.1 mM ammonium formate at pH 3.5.

Time [min]	Water [%]	Isopropanol/methanol (3:1, *v/v*) [%]
0	35	65
2	35	65
3	15	85
4	15	85
26	5	95
27	0	100
29	0	100
29.1	35	65
34	35	65

Table S2. Summary of the selected variables of the LC-MS dataset with surrogate minimal depth (SMD) (DT: drift time).

m/z	RT [min]	DT [ms]	SMD	*m/z*	RT [min]	DT [ms]	SMD
140.068	1.2	20.54	2.534	774.563	7.5	35.91	2.276
140.068	1.2	15.68	3.208	778.536	8.4	35.95	3.294
144.101	1.2	15.01	3.157	780.553	8.3	35.87	2.950
156.041	1.2	15.50	3.453	788.518	9.3	36.23	3.090
203.142	7.6	20.86	0.018	790.533	8.3	36.04	2.800
203.142	7.4	20.85	0.017	790.691	17.4	37.51	3.143
203.143	7.4	31.60	0.026	792.550	9.6	35.95	2.404
203.143	7.4	33.29	0.126	792.551	8.5	36.24	3.382
228.083	1.0	17.89	2.974	792.707	18.5	37.87	1.083
257.147	1.2	20.67	2.119	794.491	8.2	35.95	2.169
282.279	5.8	22.93	3.076	794.722	19.6	38.21	0.148
283.162	1.2	21.59	3.436	795.646	17.4	37.41	1.703
283.205	7.7	20.92	0.012	796.544	7.5	36.34	0.048
283.205	7.4	20.92	0.014	797.663	18.4	37.69	0.213
304.261	5.8	22.46	3.253	799.677	19.6	38.00	0.579
313.273	11.3	32.65	3.147	804.552	8.3	36.39	3.261
313.273	12.2	23.70	2.254	806.721	19.3	38.32	2.540
313.273	11.3	23.70	2.446	808.585	9.4	36.91	3.348
318.300	6.5	23.65	3.340	811.676	19.3	38.17	2.003
363.268	7.4	31.74	0.024	813.635	18.4	37.71	0.273
363.268	7.4	33.34	0.026	820.506	8.3	36.58	3.235
401.341	6.5	26.14	2.723	820.740	20.0	38.77	0.180
415.355	6.7	26.68	0.139	822.521	8.8	36.83	0.892
427.356	6.8	27.04	0.063	822.755	21.1	39.09	0.080
429.372	6.9	27.30	0.025	823.679	18.8	38.29	0.701
449.338	6.8	29.49	1.627	824.769	22.3	39.30	0.759
451.354	6.9	29.84	0.742	825.694	19.9	38.61	0.332
518.322	2.5	27.97	3.475	827.709	21.1	38.89	0.106
521.455	19.9	30.61	0.505	829.724	22.3	38.95	0.745
523.471	20.0	31.06	0.444	829.727	20.3	38.99	1.242
523.471	21.1	31.07	0.448	830.566	9.3	37.34	3.429
526.291	3.1	27.33	3.102	830.567	8.5	36.91	3.171
547.472	20.1	31.07	1.457	831.742	21.4	39.26	2.457
548.273	3.1	28.26	2.741	834.753	20.7	39.21	3.005
549.407	6.7	29.85	0.525	836.616	10.4	37.77	2.894
549.487	10.3	31.53	2.053	837.693	19.6	38.74	3.079
549.487	22.5	31.45	3.098	839.651	18.8	38.30	0.717
549.487	20.2	31.45	0.541	839.709	20.7	39.07	2.986
549.488	21.3	31.46	2.125	841.667	19.9	38.61	0.374

m/z	RT [min]	DT [ms]	SMD	*m/z*	RT [min]	DT [ms]	SMD
551.423	6.6	29.30	0.708	846.755	20.3	39.38	0.056
551.423	6.6	36.41	1.724	848.770	21.4	39.71	0.353
551.423	6.6	30.49	1.047	849.695	19.2	38.93	2.273
551.424	6.6	37.78	0.955	850.786	22.5	40.01	2.595
551.503	11.1	31.93	1.447	851.711	20.3	39.25	0.977
551.503	23.5	31.89	3.245	852.802	23.5	40.14	2.741
551.503	22.5	31.89	3.114	853.726	21.4	39.56	0.138
565.403	7.7	31.69	0.017	855.511	8.6	38.18	3.295
565.403	7.7	33.44	0.015	855.742	22.5	39.81	2.177
565.403	7.4	31.69	0.014	857.756	23.5	39.79	3.342
565.404	7.4	33.48	0.013	861.693	19.0	39.17	2.856
569.434	6.5	36.26	1.735	861.789	23.8	40.23	1.985
569.434	6.6	38.41	0.321	865.667	19.2	38.93	3.143
570.354	3.7	29.58	3.295	867.683	20.3	39.25	0.663
577.516	20.0	32.21	0.613	868.739	18.6	39.42	3.276
577.518	21.4	32.26	3.105	869.583	7.4	35.83	0.033
577.519	22.7	32.28	3.134	869.698	21.4	39.56	2.477
577.519	11.4	32.38	2.971	869.791	25.5	40.38	3.470
579.534	23.7	32.65	3.371	870.522	8.3	37.27	3.016
579.534	12.3	32.76	2.871	870.755	19.7	39.73	2.875
585.411	7.0	34.32	3.302	871.713	22.5	39.82	3.082
587.384	7.4	41.96	0.015	873.695	18.6	39.30	2.647
587.385	7.7	33.53	0.029	875.710	19.6	39.59	2.486
587.385	7.5	34.79	0.049	882.753	19.3	39.88	2.212
587.385	7.4	33.38	0.028	884.769	20.4	40.19	0.447
587.385	7.6	34.79	0.045	886.785	21.4	40.42	2.925
587.387	7.4	29.91	0.013	887.709	19.3	39.77	2.086
589.400	6.7	33.58	1.677	889.669	18.6	39.29	2.102
589.479	10.3	31.53	2.374	889.723	20.4	40.07	0.800
591.495	11.1	31.56	1.825	891.739	21.4	40.31	2.772
592.335	3.7	30.41	3.200	894.520	8.3	37.94	3.179
595.373	7.4	29.76	0.030	894.754	19.0	40.05	2.474
595.529	11.3	32.77	3.107	896.771	20.0	40.33	0.257
601.519	10.8	32.53	3.307	897.748	16.7	40.23	3.356
603.358	7.6	34.49	0.024	898.786	21.1	40.58	0.730
612.556	11.3	32.83	3.174	899.710	19.0	39.92	1.470
614.571	12.2	33.05	3.032	900.802	22.2	40.82	3.463
619.527	12.2	32.50	3.034	901.726	20.0	40.20	0.141
625.442	8.2	31.45	2.673	902.814	18.8	41.50	3.414
633.580	25.1	33.86	3.379	903.742	21.1	40.44	0.378

m/z	RT [min]	DT [ms]	SMD	*m/z*	RT [min]	DT [ms]	SMD
645.543	12.5	33.42	3.058	904.830	19.9	41.81	0.848
671.558	12.8	33.96	3.157	905.758	22.2	40.69	2.840
673.572	13.9	34.29	2.443	915.683	19.0	39.92	1.321
677.555	8.2	35.06	2.321	916.831	23.7	41.51	3.203
690.505	8.7	33.68	1.925	917.697	20.0	40.20	0.159
703.575	8.6	35.59	3.067	920.769	20.3	41.00	2.229
704.521	8.2	34.51	1.962	920.822	18.3	41.23	3.063
706.537	8.7	34.90	2.144	922.785	21.3	41.24	3.245
712.487	8.7	34.27	2.698	922.786	21.4	41.28	0.389
719.568	8.3	36.46	3.331	925.724	20.1	40.84	3.133
722.548	8.6	27.59	3.147	925.778	18.1	41.02	2.013
725.558	8.6	35.74	3.181	926.816	23.2	41.62	3.387
730.539	8.3	35.00	3.147	927.741	21.4	41.12	0.586
732.554	8.8	35.28	1.750	931.771	23.2	41.47	3.322
738.550	8.3	35.51	1.895	943.695	19.7	40.58	2.725
740.521	8.8	34.72	3.158	969.711	20.0	41.17	0.791
754.535	8.8	28.47	1.759	971.727	21.1	41.42	0.677
754.536	8.8	35.56	2.027	973.742	22.2	41.63	3.151
762.503	8.8	35.43	3.398	1008.893	25.3	43.59	3.196
764.519	8.3	35.73	3.421	1013.849	25.3	43.42	2.347
764.675	17.0	36.98	1.572	1034.908	25.5	44.09	3.451
766.537	9.3	35.39	2.600	1041.879	26.1	44.07	2.696
766.691	18.2	37.35	0.971	1266.200	12.4	50.72	2.816
768.551	8.4	35.68	2.117	1680.427	20.3	58.84	1.284
768.706	19.4	37.65	1.814	1684.458	21.4	59.02	1.520
769.630	17.0	36.86	1.275	1688.489	22.5	59.13	2.917
771.646	18.2	37.14	0.831				

Table S3: Summary of identification parameters of marker compounds that were selected by SMD.

Tentative Metabolite	Increased concentration in	SMD	Sum formula	Ion	*m/z* (analytical)	*m/z* (calculated)	*m/z* difference [ppm]	RT [min]	DT [ms]	CCS-value [analytical, Å]	CCS-value [LipidCCS database, Å]	CCS-value [difference, %]	Main fragments [*m/z*]
**Canthaxanthin**	Conventional	0.013	$C_{40}H_{52}O_2$	H^+	565.403	565.404	−2.49	7.7	31.69	253.60	No entry	/	363.27<br>203.14<br>133.06
**Canthaxanthin**	Conventional	0.014	$C_{40}H_{52}O_2$	H^+	565.403	565.404	−2.49	7.7	33.44	267.98	No entry	/	363.27<br>203.14<br>133.06
**Canthaxanthin**	Conventional	0.015	$C_{40}H_{52}O_2$	H^+	565.403	565.404	−2.49	7.4	31.69	253.70	No entry	/	363.27<br>203.14<br>133.06
**Canthaxanthin**	Conventional	0.017	$C_{40}H_{52}O_2$	H^+	565.403	565.404	−2.49	7.4	33.48	268.27	No entry	/	363.27<br>203.14<br>133.06
**Lutein/Zeaxanthin**	Organic	1.735	$C_{40}H_{56}O_2$	H^+	569.434	569.435	−2.48	6.5	36.26	291.49	No entry	/	551.42<br>513.29<br>371.23
**Lutein/Zeaxanthin**	Organic	0.321	$C_{40}H_{56}O_2$	H^+	569.434	569.435	−2.48	6.5	38.41	307.85	No entry	/	551.42<br>513.29<br>371.23
**DAG (16:0/16:0/0:0)**	Conventional	1.825	$C_{35}H_{68}O_5$	Na^+	591.495	591.496	−1.75	11.1	31.56	252.40	260.6	3.1	313.27<br>264.27
**PE (32:1)**	Conventional	1.925	$C_{37}H_{72}NO_8P$	H^+	690.505	690.507	−2.08	8.7	33.68	268.64	269.8<br>PE (16:1/16:0)	0.4	184.07
**TAG (16:1/16:0/14:0)**	Conventional	0.148	$C_{49}H_{92}O_6$	NH_4^+	794.722	794.723	−1.05	19.6	38.21	304.26	303.7	−0.2	549.49<br>521.46

**Tentative Metabolite**	**Increased concentration in**	**SMD**	**Sum formula**	**Ion**	***m/z* (analytical)**	***m/z* (calculated)**	***m/z* difference [ppm]**	**RT [min]**	**DT [ms]**	**CCS-value [analytical, Å]**	**CCS-value [LipidCCS database, Å]**	**CCS-value [difference, %]**	**Main fragments [*m/z*]**
**TAG (46:2)**	Conventional	0.213	$C_{49}H_{90}O_6$	Na^+	797.663	797.663	−0.6	18.4	37.69	300.11	301.4<br>TAG (16:1/16:1/14:0)	0.4	597.48<br>575.50<br>569.45<br>549.48<br>547.47<br>543.44<br>541.42<br>521.46<br>519.44<br>494.44
**TAG (48:2)**	Conventional	0.180	$C_{51}H_{94}O_6$	NH_4^+	820.740	820.739	0.91	20.0	38.77	308.50	307.2<br>TAG (16:1/16:1/16:0)	−0.4	577.52<br>575.50<br>549.49<br>547.47<br>523.47<br>521.46
**TAG (18:1/16:0/14:0)**	Conventional	0.080	$C_{51}H_{96}O_6$	NH_4^+	822.755	822.755	0.85	21.1	39.09	311.07	309.7	−0.4	577.518<br>549.487<br>523.472
**TAG (48:3)**	Conventional	0.701	$C_{51}H_{92}O_6$	Na^+	823.679	823.679	0.24	18.8	38.29	304.68	304.9<br>TAG (16:1/16:1/16:1)	0.1	597.49<br>595.47<br>575.50<br>569.45<br>567.44<br>545.45<br>543.44
**TAG (14:0/16:0/18:0)**	Conventional	0.759	$C_{51}H_{98}O_6$	NH_4^+	824.769	824.770	−0.95	22.3	39.30	312.81	312.0	−0.3	579.54<br>551.50<br>523.47

Tentative Metabolite	Increased concentration in	SMD	Sum formula	Ion	*m/z* (analytical)	*m/z* (calculated)	*m/z* difference [ppm]	RT [min]	DT [ms]	CCS-value [analytical, Å]	CCS-value [LipidCCS database, Å]	CCS-value [difference, %]	Main fragments [*m/z*]
**TAG (48:2)**	Conventional	0.332	$C_{51}H_{94}O_6$	Na^+	825.694	825.694	0.17	19.9	38.61	307.24	307.6 TAG (16:1/16:1/16:0)	0.1	597.48 569.45 547.47 523.47
**TAG (16:1/16:0/16:0)**	Conventional	0.106	$C_{51}H_{96}O_6$	Na^+	827.709	827.710	−0.76	21.1	38.89	309.46	310.1	0.2	571.47 549.48
**TAG (16:0/16:0/16:0)**	Conventional	1.242	$C_{51}H_{98}O_6$	Na^+	829.727	829.726	1.16	20.3	38.99	310.03	312.5	0.8	573.484 551.501
**TAG (18:1/16:1/16:1)**	Conventional	0.056	$C_{53}H_{96}O_6$	NH_4^+	846.755	846.755	0.10	20.3	39.38	313.08	311.0	−0.7	575.50 573.49 549.49 547.47
**TAG (18:1/16:1/16:0)**	Conventional	0.353	$C_{53}H_{98}O_6$	NH_4^+	848.770	848.770	0.28	21.4	39.71	315.75	313.4	−0.7	575.5 551.50 549.49
**TAG (18:1/16:0/16:0)**	Conventional	2.595	$C_{53}H_{100}O_6$	NH_4^+	850.786	850.786	0.70	22.5	40.01	318.09	315.7	−0.8	577.52 551.50
**TAG (18:1/16:1/16:1)**	Conventional	0.977	$C_{53}H_{96}O_6$	Na^+	851.711	851.710	1.07	20.3	39.25	312.02	311.3	−0.2	597.48 595.47 575.50 573.49 571.47 549.49
**TAG (18:1/16:1/16:0)**	Conventional	0.138	$C_{53}H_{98}O_6$	Na^+	853.726	853.726	1.01	21.4	39.56	314.54	313.8	−0.2	597.48 575.50 571.47 549.49

Tentative Metabolite	Increased concentration in	SMD	Sum formula	Ion	*m/z* (analytical)	*m/z* (calculated)	*m/z* difference [ppm]	RT [min]	DT [ms]	CCS-value [analytical, Å]	CCS-value [LipidCCS database, Å]	CCS-value [difference, %]	Main fragments [*m/z*]
**TAG (18:1/16:0/16:0)**	Conventional	2.177	$C_{53}H_{100}O_6$	Na^+	855.742	855.741	0.35	22.5	39.81	316.54	316.1	−0.1	599.50<br>577.52<br>573.48<br>551.50
**TAG (18:1/18:0/16:0)**	Conventional	1.985	$C_{55}H_{104}O_6$	H^+	861.789	861.791	−1.47	23.8	40.23	319.91	320.1	0.1	605.55<br>579.53<br>577.52
**TAG (53:5)**	Organic	0.447	$C_{56}H_{98}O_6$	NH_4^+	884.769	884.770	−1.92	20.4	40.19	319.32	319.4<br>TAG (17:0/18:2/18:3)	0.0	599.51<br>587.50<br>585.48
**TAG (18:3/18:2/18:1)**	Organic	0.257	$C_{57}H_{98}O_6$	NH_4^+	896.771	896.770	0.49	20.0	40.33	320.46	319.4	−0.3	601.52<br>599.50<br>597.49
**TAG (18:2/18:2/18:1)**	Organic	0.730	$C_{57}H_{100}O_6$	NH_4^+	898.786	898.786	0.66	21.1	40.58	322.50	320.7	−0.6	601.52<br>599.50
**TAG (18:3/18:2/18:1)**	Organic	0.141	$C_{57}H_{98}O_6$	Na^+	901.726	901.726	−0.27	20.0	40.20	319.47	319.7	0.1	623.49<br>621.48<br>619.47<br>601.52<br>599.50<br>597.49
**TAG (18:2/18:2/18:1)**	Organic	0.378	$C_{57}H_{100}O_6$	Na^+	903.742	903.741	0.67	21.1	40.44	321.40	321.0	−0.1	623.49<br>621.48<br>601.52<br>599.50
**TAG (56:7)**	Organic	0.389	$C_{59}H_{100}O_6$	NH_4^+	922.786	922.786	−0.24	21.4	41.28	327.78	325.5<br>TAG (18:3/18:3/20:1)	−0.7	854.73<br>651.53<br>625.52<br>577.52

**Tentative Metabolite**	**Increased concentration in**	**SMD**	**Sum formula**	**Ion**	***m/z* (analytical)**	***m/z* (calculated)**	***m/z* difference [ppm]**	**RT [min]**	**DT [ms]**	**CCS-value [analytical, Å]**	**CCS-value [LipidCCS database, Å]**	**CCS-value [difference, %]**	**Main fragments [*m/z*]**
**TAG (56:8)**	Organic	3.133	$C_{59}H_{98}O_6$	Na^+	925.724	925.724	−1.29	20.1	40.84	324.70	324.4<br>TAG (18:3/18:3/20:2	−0.1	669.48<br>621.48<br>607.50<br>599.50<br>597.49
**TAG (56:7)**	Organic	0.586	$C_{59}H_{100}O_6$	Na^+	927.741	927.741	−0.68	21.4	41.12	326.55	325.8	−0.2	673.51<br>647.50<br>625.52<br>599.50
**TAG (56:5)**	Organic	3.322	$C_{59}H_{104}O_6$	Na+	931.771	931.773	−1.55	23.2	41.47	329.37	328.1<br>TAG (18:2/18:2/20:0)	−0.4	677.55<br>675.53<br>651.53<br>649.52<br>647.50<br>631.56<br>629.55<br>627.53<br>605.55<br>603.53<br>601.52<br>525.52

Table S4. Confusion matrix of the random forest for the differentiation of the husbandries of the 270 egg samples based on photometry data in percentage.

		Predicted		
		Organic (%)	Conventional (%)	Sensitivity (%)
True	Organic (%)	88.0	12.0	88.0
	Conventional (%)	5.6	94.4	94.4
	Specifity (%)	94.0	88.7	92.2

Table S5. Summary of the selected variables of the FT-NIR spectroscopy dataset with SMD.

Wavenumber [cm^{-1}]	SMD	Wavenumber [cm^{-1}]	SMD	Wavenumber [cm^{-1}]	SMD
3963	1.100	5249	1.019	6055	0.052
4034	1.075	5259	0.979	6065	0.037
4198	0.014	5269	0.990	6075	0.062
4218	0.089	5279	1.085	6085	0.091
4238	0.631	5289	1.054	6095	0.180
4259	0.588	5300	1.012	6106	0.467
4269	1.011	5310	1.031	6116	0.295
4279	0.844	5320	1.072	6126	0.242
4300	0.516	5330	0.874	6136	0.634
4310	0.608	5340	0.747	6146	0.769
4330	0.525	5351	0.864	6157	1.012
4340	0.912	5361	1.016	6197	1.143
4381	0.741	5371	1.199	6208	1.015
4391	0.669	5381	0.967	6350	0.648
4565	1.074	5412	0.799	6361	0.239
4575	1.247	5422	0.824	6371	0.282
4636	0.859	5483	0.553	6381	0.348
4647	0.717	5493	0.210	6391	0.404
4667	0.302	5504	0.450	6453	1.214
4677	0.577	5534	0.779	6463	1.053
4708	0.933	5544	0.137	6626	0.632
4718	0.560	5555	0.221	6657	1.052
4728	0.347	5565	0.269	6687	1.038
4738	0.615	5575	0.238	6697	0.756
4749	0.695	5585	0.262	6708	0.821
4759	0.790	5595	0.514	6718	0.940
4769	0.741	5606	0.790	6728	0.859
4922	1.208	5616	0.405	6738	0.823
4932	0.964	5626	0.644	6748	0.899
4942	0.876	5667	0.371	6759	1.157
4953	0.805	5677	1.001	6912	1.242

Wavenumber [cm⁻¹]	SMD	Wavenumber [cm⁻¹]	SMD	Wavenumber [cm⁻¹]	SMD
4963	0.870	5718	0.175	6952	1.154
4973	0.928	5728	0.869	6963	1.204
4983	0.858	5779	0.423	7024	1.002
4993	0.774	5789	0.244	7034	1.173
5004	0.849	5800	0.330	7044	1.159
5014	0.855	5810	0.390	7054	1.093
5024	0.883	5820	0.547	7065	1.214
5034	0.888	5830	1.074	7157	1.003
5044	0.959	5871	0.197	7167	0.650
5055	0.859	5881	0.051	7177	0.302
5065	0.834	5891	0.021	7187	0.989
5075	0.909	5902	0.054	7259	0.980
5085	0.827	5912	0.098	7269	0.556
5095	0.811	5922	0.061	7279	0.527
5106	0.909	5932	0.044	7289	0.799
5116	0.803	5942	0.029	7299	1.113
5126	1.022	5953	0.029	7310	1.112
5136	0.971	5963	0.042	7320	1.238
5167	0.886	5973	0.449	7330	0.908
5187	1.240	6004	0.336	8197	0.888
5198	0.990	6014	0.019	8218	0.745
5208	0.928	6024	0.016	8228	1.230
5218	0.966	6034	0.037	8412	1.076
5228	0.966	6044	0.079	8544	1.246
5238	0.958				

Table S6. Confusion matrix of the random forest for the differentiation of the husbandries of the 270 egg samples based on the merged LC-MS and FT-NIR spectroscopy data in percentage.

		Predicted		
		Organic (%)	Conventional (%)	Sensitivity (%)
True	Organic (%)	97.8	2.2	97.8
	Conventional (%)	4.5	95.5	95.5
	Specifity (%)	91.8	98.8	96.3

Table S7: Summary of the selected LC-MS variables from the merged LC-MS and FT-NIR spectroscopy dataset with SMD.

m/z	RT [min]	DT [ms]	SMD	*m/z*	RT [min]	DT [ms]	SMD
59.072	1.2	14.00	1.763	765.526	8.4	28.27	1.479
96.078	1.2	15.64	1.376	766.537	9.3	35.39	0.054
102.127	19.7	41.75	1.926	766.691	18.2	37.35	0.036
109.075	1.4	13.58	0.407	768.551	8.4	35.68	0.057
118.086	1.2	14.07	1.796	768.706	19.4	37.65	0.070
140.068	1.2	20.54	0.133	769.630	17.0	36.86	0.049
140.068	1.2	15.68	0.646	770.567	8.8	35.94	1.122
144.101	1.2	15.01	0.121	771.646	18.2	37.14	0.034
146.059	1.3	15.00	1.244	772.584	9.4	36.22	1.661
156.041	1.2	15.50	0.545	774.503	8.2	35.83	1.688
158.117	1.2	15.51	0.484	774.563	7.5	35.91	0.071
166.084	1.2	16.67	1.965	778.536	8.4	35.95	0.270
168.113	1.4	15.89	0.697	778.572	10.3	36.04	0.953
188.070	1.2	16.81	1.927	780.550	12.1	36.16	1.852
203.142	7.6	20.86	0.001	780.553	8.3	35.87	0.086
203.142	7.4	20.85	0.001	786.503	8.8	35.93	2.075
203.143	7.4	31.60	0.008	787.667	11.8	37.96	2.048
203.143	7.4	33.29	0.029	788.518	9.3	36.23	0.162
228.083	1.0	17.89	0.359	790.533	8.3	36.04	0.060
244.078	1.2	18.40	0.852	790.691	17.4	37.51	0.426
254.247	4.0	21.98	1.572	792.550	9.6	35.95	0.047
256.263	5.3	22.09	1.377	792.551	8.5	36.24	0.127
257.147	1.2	20.67	0.092	792.707	18.5	37.87	0.051
280.263	4.6	22.59	0.975	793.544	8.6	37.11	0.939
282.279	5.8	22.93	0.119	794.491	8.2	35.95	0.402
282.279	5.8	21.97	0.787	794.722	19.6	38.21	0.015
283.162	1.2	21.59	0.214	795.571	10.2	36.26	0.499
283.205	7.7	20.92	0.001	795.646	17.4	37.41	0.058
283.205	7.4	20.92	0.001	796.544	7.5	36.34	0.011
284.294	6.5	23.05	1.599	797.663	18.4	37.69	0.021
302.245	4.6	21.79	0.927	799.677	19.6	38.00	0.025
304.261	5.8	22.46	0.203	800.518	8.3	36.25	0.358
311.257	10.4	22.95	0.332	800.615	10.5	37.05	0.308
313.273	12.2	32.84	0.247	802.535	8.3	36.22	0.430
313.273	5.6	23.76	1.757	804.550	9.2	36.69	0.645
313.273	11.3	32.65	0.062	804.552	8.3	36.39	0.102
313.273	12.2	23.70	0.038	806.568	9.2	36.69	1.411
313.273	11.3	23.70	0.030	806.569	8.8	36.63	0.685
318.300	6.5	23.65	0.218	806.721	19.3	38.32	0.323

m/z	RT [min]	DT [ms]	SMD	*m/z*	RT [min]	DT [ms]	SMD
320.233	5.8	23.02	0.582	808.585	9.4	36.91	0.275
337.273	10.8	23.52	1.839	809.661	18.1	37.86	1.935
339.288	10.8	32.59	0.727	811.676	19.3	38.17	0.092
339.289	10.8	24.08	0.990	812.613	10.4	37.24	0.229
339.289	12.5	33.51	0.193	813.635	18.4	37.71	0.020
339.289	10.4	24.07	1.540	816.550	10.2	37.12	1.021
339.289	12.5	24.09	0.211	818.508	8.3	36.32	1.341
341.304	12.1	24.67	0.700	818.724	18.8	38.42	0.851
341.304	13.4	24.69	0.405	819.645	17.3	37.99	1.605
341.304	6.5	24.78	1.160	820.506	8.3	36.58	0.863
341.304	12.5	24.68	0.224	820.740	20.0	38.77	0.015
341.305	12.5	33.54	0.227	821.662	17.8	38.02	1.933
341.305	12.1	33.04	1.664	822.521	8.8	36.83	0.011
344.261	1.4	24.51	1.703	822.635	11.5	37.95	0.487
351.250	4.1	23.27	1.079	822.755	21.1	39.09	0.010
353.266	5.6	23.72	0.942	823.679	18.8	38.29	0.030
363.268	7.4	31.74	0.006	824.769	22.3	39.30	0.019
363.268	7.4	33.34	0.008	825.694	19.9	38.61	0.016
367.224	4.1	23.72	1.642	826.535	8.3	36.77	1.752
367.334	6.5	25.21	1.251	826.571	11.1	37.45	1.700
369.239	5.6	24.27	0.408	827.709	21.1	38.89	0.011
369.351	8.3	25.29	1.297	829.724	22.3	38.95	0.021
371.264	8.6	20.35	0.473	829.727	20.3	38.99	0.020
381.297	6.5	24.84	0.183	830.566	9.3	37.34	0.333
397.271	6.5	25.34	0.516	830.567	8.5	36.91	0.133
399.324	6.0	26.62	1.732	831.742	21.4	39.26	0.088
400.341	3.3	26.45	1.001	832.738	19.6	38.86	2.021
401.341	6.5	26.14	0.176	833.758	22.5	39.51	0.364
407.313	6.7	25.49	1.715	834.677	12.3	38.29	1.558
409.328	7.1	25.87	0.855	834.753	20.7	39.21	0.336
415.355	6.7	26.68	0.024	836.614	11.2	37.84	1.480
423.322	6.5	28.76	0.671	836.616	10.4	37.77	0.054
425.265	4.7	24.73	1.394	837.635	17.8	38.01	1.215
425.339	7.1	27.35	1.235	837.693	19.6	38.74	0.414
427.281	5.8	25.26	0.447	838.628	12.9	37.99	1.794
427.356	6.8	27.04	0.014	839.651	18.8	38.30	0.015
429.372	6.9	27.30	0.003	839.709	20.7	39.07	0.245
437.338	6.7	29.29	0.829	840.641	11.7	38.03	0.565
441.238	4.7	25.07	0.464	841.667	19.9	38.61	0.015
449.338	6.8	29.49	0.041	842.492	8.2	36.55	0.868

m/z	RT [min]	DT [ms]	SMD	m/z	RT [min]	DT [ms]	SMD
451.354	6.9	29.84	0.016	842.723	18.3	38.78	2.043
482.323	2.9	28.15	0.555	844.616	11.4	38.34	0.959
504.305	2.9	28.55	0.201	844.616	11.9	38.31	0.463
504.306	5.4	28.33	1.899	846.755	20.3	39.38	0.006
508.338	3.2	28.60	1.005	847.679	18.2	38.67	1.786
510.355	4.3	29.18	1.209	848.770	21.4	39.71	0.014
518.322	2.5	27.97	0.176	849.695	19.2	38.93	0.060
521.455	18.8	30.63	1.774	850.784	23.3	39.90	0.715
521.455	19.9	30.61	0.022	850.786	22.5	40.01	0.088
523.471	19.9	31.06	0.380	851.711	20.3	39.25	0.029
523.471	20.0	31.06	0.019	852.802	23.5	40.14	0.097
523.471	21.1	31.07	0.021	853.724	22.0	39.48	1.241
526.286	5.4	28.54	1.444	853.726	19.7	38.77	1.306
526.291	3.1	27.33	0.106	853.726	21.4	39.56	0.009
528.306	3.8	27.75	0.236	855.511	8.6	38.18	0.161
530.320	3.2	28.94	0.719	855.703	16.3	39.18	1.729
532.337	4.3	29.49	1.058	855.742	22.5	39.81	0.054
538.386	6.1	30.17	1.719	856.737	19.1	39.30	0.745
540.304	2.5	28.69	0.179	857.756	23.5	39.79	0.120
542.322	3.0	29.08	1.504	858.596	10.4	38.17	0.237
547.472	20.1	31.07	0.038	858.754	20.0	39.52	1.551
548.273	3.1	28.26	0.063	860.770	21.1	39.77	1.862
548.310	4.2	29.69	0.639	861.693	19.0	39.17	0.089
548.370	4.4	29.33	1.915	861.789	23.8	40.23	0.017
549.407	6.7	29.85	0.018	862.785	22.2	40.09	0.455
549.487	19.8	31.44	1.390	864.763	17.0	39.84	1.467
549.487	10.3	31.53	0.073	864.763	16.7	39.81	0.806
549.487	22.5	31.45	0.158	864.800	23.3	40.35	0.176
549.487	20.1	31.45	1.696	865.667	19.2	38.93	0.232
549.487	20.2	31.45	0.013	866.815	24.3	40.50	0.802
549.488	21.3	31.46	0.060	867.683	20.3	39.25	0.016
551.419	6.6	34.12	0.814	867.739	22.2	39.96	0.647
551.423	6.6	29.30	0.029	868.505	8.2	37.46	1.779
551.423	6.6	36.41	0.098	868.739	18.6	39.42	0.617
551.423	6.6	30.49	0.029	869.583	7.4	35.83	0.011
551.424	6.6	37.78	0.038	869.698	21.4	39.56	0.056
551.502	21.4	31.88	1.560	869.719	16.5	39.62	2.038
551.503	11.1	31.93	0.046	869.719	17.0	39.67	0.924
551.503	23.5	31.89	0.124	869.755	23.3	40.15	0.825
551.503	22.5	31.89	0.167	869.791	25.5	40.38	0.238

m/z	RT [min]	DT [ms]	SMD	*m/z*	RT [min]	DT [ms]	SMD
560.367	6.1	30.43	1.244	870.522	8.3	37.27	0.097
563.538	24.5	32.32	0.371	870.755	19.7	39.73	0.367
565.403	7.7	31.69	0.001	871.677	17.7	39.03	0.499
565.403	7.7	33.44	0.001	871.678	18.2	39.29	1.733
565.403	7.4	31.69	0.001	871.713	22.5	39.82	0.163
565.404	7.4	33.48	0.001	873.695	18.6	39.30	0.151
568.339	3.0	29.23	0.386	875.710	19.6	39.59	0.127
569.434	6.5	36.26	0.092	876.802	23.4	40.51	0.529
569.434	6.6	38.41	0.013	877.726	20.7	39.85	1.206
570.354	3.7	29.58	0.163	878.583	14.3	38.29	1.806
573.487	20.4	31.53	1.857	878.585	11.1	38.45	1.922
575.503	20.4	31.92	0.349	878.817	23.8	40.82	1.892
575.503	21.5	31.91	0.518	879.739	20.0	39.42	0.806
577.516	20.0	32.21	0.027	880.833	24.7	40.93	0.239
577.518	21.4	32.26	0.186	882.753	19.3	39.88	0.042
577.518	23.3	32.27	0.733	883.734	17.1	40.00	1.039
577.519	22.7	32.28	0.063	883.772	24.4	40.52	0.771
577.519	11.4	32.38	0.117	884.769	20.4	40.19	0.010
579.534	23.7	32.65	0.141	885.785	25.2	40.54	0.877
579.534	24.7	32.67	0.375	885.788	24.7	40.59	0.356
579.534	12.3	32.76	0.098	886.746	15.0	39.76	1.163
585.411	7.0	34.32	0.243	886.785	21.4	40.42	0.059
587.384	7.4	41.96	0.001	887.709	19.3	39.77	0.051
587.385	7.7	33.53	0.007	888.802	22.4	40.69	1.643
587.385	7.5	34.79	0.009	889.669	18.6	39.29	0.232
587.385	7.4	33.38	0.004	889.723	20.4	40.07	0.021
587.385	7.6	34.79	0.010	890.827	24.9	40.86	1.644
587.387	7.4	29.91	0.001	891.703	14.9	39.64	0.829
589.400	6.7	33.58	0.092	891.739	21.4	40.31	0.038
589.479	10.3	31.53	0.098	892.793	17.5	40.54	1.973
590.321	3.0	29.91	0.306	892.794	17.8	40.61	0.191
591.495	11.1	31.56	0.049	893.756	22.4	40.56	0.729
591.533	11.9	32.79	1.331	894.520	8.3	37.94	0.117
592.335	3.7	30.41	0.136	894.754	19.0	40.05	0.052
593.512	10.5	32.31	1.606	894.846	25.3	41.28	1.121
595.373	7.4	29.76	0.007	895.730	14.7	40.00	1.536
595.529	11.3	32.77	0.046	896.771	20.0	40.33	0.008
599.502	10.1	32.17	1.384	897.748	16.7	40.23	0.331
601.385	7.0	34.91	0.927	897.750	17.8	40.42	1.421
601.519	10.8	32.53	0.103	897.823	26.2	41.14	0.363

m/z	RT [min]	DT [ms]	SMD	*m/z*	RT [min]	DT [ms]	SMD
603.358	7.6	34.49	0.002	898.786	21.1	40.58	0.016
605.551	12.6	33.19	0.546	899.710	19.0	39.92	0.018
607.565	24.9	33.42	1.335	900.802	22.2	40.82	0.329
607.565	25.8	33.47	2.056	901.726	20.0	40.20	0.007
607.565	13.6	33.54	0.214	901.762	24.7	40.72	1.459
612.556	11.3	32.83	0.069	902.814	18.8	41.50	0.296
614.571	12.2	33.05	0.053	902.818	23.3	41.09	0.679
617.512	11.3	32.52	0.286	903.742	21.1	40.44	0.008
617.512	10.1	32.26	0.835	904.830	19.9	41.81	0.031
619.527	12.2	32.50	0.111	905.758	22.2	40.69	0.045
619.529	10.8	32.75	0.444	907.734	16.4	40.21	1.693
623.560	12.5	33.65	0.178	907.774	23.2	40.95	0.652
625.442	8.2	31.45	0.104	908.863	25.8	41.67	1.467
631.527	11.9	32.97	1.768	909.711	14.2	40.05	1.788
631.563	13.9	33.40	1.974	909.789	24.2	41.19	0.967
631.565	12.8	33.71	0.565	911.729	15.1	40.39	1.523
632.634	13.8	34.85	0.823	913.669	18.0	39.61	0.221
633.484	11.3	32.59	1.272	913.720	16.8	40.27	1.898
633.580	25.1	33.86	0.156	913.819	25.8	41.36	1.785
634.539	10.1	32.28	1.740	915.683	19.0	39.92	0.030
635.500	12.2	32.77	0.149	915.702	13.1	39.92	1.281
636.556	10.8	32.74	0.337	916.831	23.7	41.51	0.071
639.494	10.2	32.31	2.001	917.697	20.0	40.20	0.010
640.588	12.5	33.72	0.289	917.719	13.9	40.13	1.340
641.512	10.8	32.68	0.474	919.713	21.1	40.46	0.269
642.602	13.4	33.85	0.254	919.735	16.2	40.48	0.447
644.596	12.4	33.93	0.675	920.769	20.3	41.00	0.089
645.543	12.5	33.42	0.053	920.822	18.3	41.23	0.148
647.559	13.5	33.39	0.181	920.862	25.5	41.86	1.753
650.642	13.1	35.03	1.718	921.713	21.4	40.61	0.385
650.645	13.8	35.07	1.254	921.786	23.7	41.39	1.697
657.483	10.9	32.63	0.988	922.785	21.3	41.24	0.071
659.557	13.2	33.86	1.500	922.786	21.4	41.28	0.006
660.555	10.7	33.33	0.986	923.729	22.5	41.08	0.660
660.568	12.4	34.21	0.727	923.745	23.3	40.95	2.021
660.568	12.4	34.21	0.743	923.802	24.7	41.56	0.198
660.624	13.5	34.57	2.012	925.724	20.1	40.84	0.081
661.516	12.5	33.47	1.583	925.778	18.1	41.02	0.030
662.570	11.2	33.49	1.324	926.816	23.2	41.62	0.218
663.532	13.5	33.61	0.213	927.703	15.1	40.49	1.950

m/z	RT [min]	DT [ms]	SMD	*m/z*	RT [min]	DT [ms]	SMD
666.482	8.6	33.44	1.383	927.741	21.4	41.12	0.009
666.601	12.8	34.16	0.139	928.832	23.5	41.69	1.743
671.558	12.8	33.96	0.117	930.846	20.3	42.33	0.618
673.572	13.9	34.29	0.032	931.770	22.5	41.37	1.406
677.555	8.2	35.06	0.107	931.771	23.2	41.47	0.102
685.497	11.3	34.21	0.412	932.790	15.4	41.01	1.008
686.605	12.0	35.06	1.844	932.864	25.2	42.05	0.848
687.531	12.8	33.97	1.486	933.786	23.5	41.56	0.731
688.490	8.2	33.45	0.442	934.877	22.5	42.79	1.929
690.505	8.7	33.68	0.045	934.878	26.0	42.21	0.493
693.540	12.1	34.49	0.581	937.724	17.1	40.71	0.520
693.541	11.7	34.36	1.211	939.833	26.0	42.02	0.783
695.556	12.5	34.62	0.728	940.829	19.7	42.24	1.420
701.559	8.2	35.32	1.212	941.847	26.4	42.08	1.895
703.572	8.3	35.59	1.402	943.695	19.7	40.58	0.281
703.575	8.6	35.59	0.126	944.768	19.6	41.31	0.207
704.521	8.2	34.51	0.189	945.712	20.7	40.87	1.761
704.556	10.2	34.57	1.746	948.798	21.4	41.77	0.835
706.537	8.7	34.90	0.038	949.723	19.5	41.22	1.284
712.487	8.7	34.27	0.165	953.773	24.7	42.06	0.207
713.529	12.5	35.14	0.450	958.877	25.5	42.47	1.662
714.506	8.3	33.82	0.275	964.923	24.7	43.70	0.332
714.558	8.6	27.83	0.898	967.865	26.4	42.65	1.948
716.521	8.9	34.22	0.970	969.711	20.0	41.17	0.014
719.568	8.3	36.46	0.151	971.727	21.1	41.42	0.012
722.548	8.6	27.59	0.221	973.742	22.2	41.63	0.088
725.558	8.6	35.74	0.056	975.758	23.3	41.88	0.513
726.055	8.6	27.99	0.919	980.862	20.0	43.18	0.222
726.504	8.2	34.75	1.463	1006.878	24.5	43.46	1.356
730.539	8.3	35.00	0.851	1008.893	25.3	43.59	0.085
732.553	9.3	35.26	0.939	1013.849	25.3	43.42	0.046
732.554	8.8	35.28	0.048	1015.863	25.9	43.48	0.241
734.568	10.2	35.66	1.412	1027.667	5.4	41.61	1.901
734.569	9.4	35.70	1.037	1034.908	25.5	44.09	0.141
736.487	8.3	34.46	1.917	1039.863	25.5	43.92	0.178
738.503	8.9	34.79	0.387	1041.879	26.1	44.07	0.073
738.550	8.3	35.51	0.047	1087.731	23.8	44.32	1.807
740.521	8.8	34.72	0.073	1266.200	12.4	50.72	0.067
742.537	8.3	35.33	0.451	1292.215	12.6	51.10	1.650
744.553	8.7	35.36	1.750	1293.162	12.6	50.68	0.440

m/z	RT [min]	DT [ms]	SMD	*m/z*	RT [min]	DT [ms]	SMD
751.546	7.1	33.76	1.082	1322.262	13.8	51.90	1.983
752.519	8.4	28.41	0.915	1528.128	9.7	52.69	1.729
754.535	8.8	28.47	0.025	1540.127	9.0	53.03	0.514
754.536	8.8	35.56	0.045	1564.127	8.9	53.40	0.945
757.043	8.6	28.20	1.023	1588.125	8.9	53.73	2.016
762.503	8.8	35.43	0.130	1612.127	8.8	54.12	1.999
764.519	8.3	35.73	0.133	1680.427	20.3	58.84	0.026
764.522	8.8	35.09	1.526	1684.458	21.4	59.02	0.036
764.675	17.0	36.98	0.062	1688.489	22.5	59.13	0.148

Table S8: Summary of the selected FT-NIR spectroscopy variables from the merged LC-MS and FT-NIR spectroscopy dataset with SMD.

Wavenumber [cm^{-1}]	SMD	Wavenumber [cm^{-1}]	SMD	Wavenumber [cm^{-1}]	SMD
3963	1.920	5606	2.048	5973	1.444
4198	0.005	5616	1.164	6004	0.425
4218	0.051	5626	1.930	6014	0.035
4238	0.829	5667	0.822	6024	0.071
4259	0.971	5677	0.850	6034	0.023
4279	1.774	5708	1.439	6044	0.262
4300	0.729	5718	0.079	6055	0.051
4310	0.993	5728	0.126	6065	0.028
4330	0.695	5738	1.063	6075	0.171
4340	1.807	5748	1.466	6085	0.173
4381	1.002	5759	2.015	6095	1.040
4391	1.004	5779	0.275	6106	1.479
4565	1.863	5789	0.049	6116	0.854
4636	2.035	5800	0.189	6126	0.887
4647	1.564	5810	0.116	6361	1.327
4667	1.326	5820	0.559	6371	1.354
4728	1.908	5830	1.490	6391	0.421
5483	1.360	5871	0.613	6442	1.747
5493	0.384	5881	0.076	6453	2.043
5504	1.143	5891	0.029	6463	2.035
5514	1.693	5902	0.118	6616	1.688
5534	1.767	5912	0.358	6626	0.999
5544	0.157	5922	0.111	6657	0.506
5555	0.050	5932	0.063	6697	1.372
5565	0.252	5942	0.040	7177	1.557
5575	0.046	5953	0.055	8218	1.111

**Wavenumber [cm⁻¹]**	**SMD**	**Wavenumber [cm⁻¹]**	**SMD**	**Wavenumber [cm⁻¹]**	**SMD**
5585	0.769	5963	0.064	8534	1.240
5595	1.572				
